# 3D printing sequentially strengthening high-strength natural polymer hydrogel bilayer scaffold for cornea regeneration

**DOI:** 10.1093/rb/rbae012

**Published:** 2024-02-09

**Authors:** Xiongfeng Nie, Yong Tang, Tengling Wu, Xinrui Zhao, Ziyang Xu, Rong Yang, Yage Sun, Bin Wu, Quanhong Han, Jingwen Hui, Wenguang Liu

**Affiliations:** School of Material Science and Engineering, Tianjin Key Laboratory of Composite and Functional Materials, Tianjin University, Tianjin 300350, China; Tianjin Eye Hospital, Tianjin Key Lab of Ophthalmology and Visual Science, Tianjin Eye Institute, Tianjin 300020, China; School of Material Science and Engineering, Tianjin Key Laboratory of Composite and Functional Materials, Tianjin University, Tianjin 300350, China; School of Material Science and Engineering, Tianjin Key Laboratory of Composite and Functional Materials, Tianjin University, Tianjin 300350, China; School of Material Science and Engineering, Tianjin Key Laboratory of Composite and Functional Materials, Tianjin University, Tianjin 300350, China; School of Material Science and Engineering, Tianjin Key Laboratory of Composite and Functional Materials, Tianjin University, Tianjin 300350, China; School of Material Science and Engineering, Tianjin Key Laboratory of Composite and Functional Materials, Tianjin University, Tianjin 300350, China; Tianjin Eye Hospital, Tianjin Key Lab of Ophthalmology and Visual Science, Tianjin Eye Institute, Tianjin 300020, China; Tianjin Eye Hospital, Tianjin Key Lab of Ophthalmology and Visual Science, Tianjin Eye Institute, Tianjin 300020, China; Tianjin Eye Hospital, Tianjin Key Lab of Ophthalmology and Visual Science, Tianjin Eye Institute, Tianjin 300020, China; School of Material Science and Engineering, Tianjin Key Laboratory of Composite and Functional Materials, Tianjin University, Tianjin 300350, China

**Keywords:** 3D printing, high-strength hydrogel, natural polymer, cornea regeneration, bilayer scaffold

## Abstract

3D printing of high-strength natural polymer biodegradable hydrogel scaffolds simultaneously resembling the biomechanics of corneal tissue and facilitating tissue regeneration remains a huge challenge due to the inherent brittleness of natural polymer hydrogels and the demanding requirements of printing. Herein, concentrated aqueous solutions of gelatin and carbohydrazide-modified alginate (Gel/Alg-CDH) are blended to form a natural polymer hydrogel ink, where the hydrazides in Alg-CDH are found to form strong hydrogen bonds with the gelatin. The hydrogen-bonding-strengthened Gel/Alg-CDH hydrogel demonstrates an appropriate thickened viscosity and shear thinning for extrusion printing. The strong hydrogen bonds contribute to remarkably increased mechanical properties of Gel/Alg-CDH hydrogel with a maximum elongation of over 400%. In addition, sequentially Ca^2+^-physical crosslinking and then moderately chemical crosslinking significantly enhance the mechanical properties of Gel/Alg-CDH hydrogels that ultimately exhibit an intriguing J-shaped stress–strain curve (tensile strength of 1.068 MPa and the toughness of 677.6 kJ/m^2^). The dually crosslinked Gel-Alg-CDH-Ca^2+^-EDC hydrogels demonstrate a high transparency, physiological swelling stability and rapid enzymatic degradability, as well as suturability. The growth factor and drug-loaded biomimetic bilayer hydrogel scaffold are customized via a multi-nozzle printing system. This bioactive bilayer hydrogel scaffold considerably promotes regeneration of corneal epithelium and stroma and inhibits cornea scarring in rabbit cornea keratoplasty.

## Graphical Abstract

**Figure:**
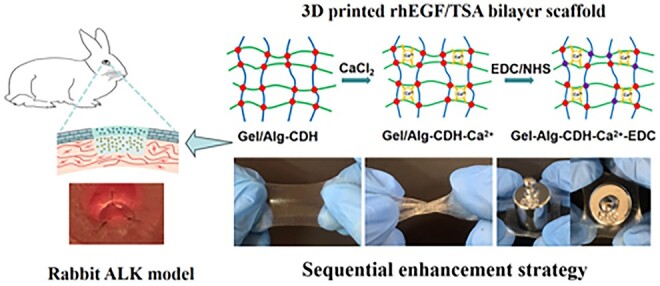

## Introduction

The cornea is a transparent part with a certain curvature in the front of the outer layer of the eyeball. The refraction of the cornea accounts for 2/3 of the total refraction of the eyeball, which is essential for vision [1]. Cornea diseases such as keratoconus, bullous keratopathy or accidental physical trauma can damage the cornea and even cause vision loss [2]. For these cornea injuries, cornea transplantation is the most common and successful surgical procedure to restore cornea transparency and vision. However, limited graft survival, disease infection, immune rejection and serious complications lead to a high failure rate of cornea transplantation, and the failure rate is even higher with longer transplant time [3, 4]. In addition, the global supply of cornea donors is seriously insufficient, and more than 12.7 million people worldwide are currently waiting for cornea transplants [5]. Therefore, tissue-engineered cornea scaffolds have become a therapeutic alternative to cornea transplantation due to their design flexibility and versatility [6, 7].

So far, various natural polymers or derivatives (e.g. decellularized corneal matrix [8], collagen [9], gelatin methacrylate (GelMA) [10], hyaluronic acid [11], chitosan [12], etc.) and synthetic polymers (e.g. poly(ethylene glycol) [13], poly-hydroxyethylmethacrylate [14], poly(vinyl alcohol) [15], poly(ε-caprolactone) (PCL) [16], etc.) have been used to construct cornea scaffolds through different fabrication methods, such as casting [17], *in situ* gelation [11], self-assembly [18], electrospinning, etc. [15, 16]. Natural cornea has a complex multilayer structure, which is mainly composed of epithelium, stroma and endothelium. Each of the three layers of the cornea is critical to maintaining normal visual function [2, 19]. However, these traditional manufacturing methods cannot simulate the complex multilayer structure of natural cornea, and can only obtain a single component of isotropic cornea scaffold. 3D printing, an emerging additive manufacturing technology, can incorporate different cells/active factors and biomaterials in a 3D geometric structure by layer-by-layer deposition, and finally customize specific cornea tissue in a flexible and automatic way [20–22]. We previously reported a 3D-printed biomimetic epithelium/stroma bilayer hydrogel scaffold, where the synergistic effect of microstructure and precisely located cells in epithelia and stroma layer provided an optimal microenvironment for cornea regeneration [23]. Other 3D printing hydrogels have also been developed to mimic the natural cornea microenvironment for cornea regeneration [24, 25]. These hydrogels are usually based on natural polymers with good biocompatibility and degradability, such as collagen, gelatin, hyaluronic acid, etc., which are the main components of the natural cornea extracellular matrix. But these natural polymer hydrogels suffer the drawback of insufficient mechanical properties to withstand surgical sutures, thus limiting the reliability of their clinical application. Synthetic polymer-reinforcement strategies have been increasingly applied to enhance the mechanical performances of natural polymer-based hydrogels [26, 27]. The mechanical properties of synthetic fiber (such as PCL [16] or poly(ε-caprolactone)-poly(ethylene glycol) (PECL ) [28]) reinforced GelMA hydrogel are significantly improved, and their tensile strength and elongation are even much higher than those of natural cornea. Notably, these synthetic fiber-strengthened hydrogel scaffolds can allow direct surgical suture. However, these synthetic fibers usually cannot be directly introduced into a 3D printing system, and the synthetic polymer grid in the natural hydrogel scaffold was not degraded *in vivo* even after 3 months, which negatively affected the ingrowth of neotissue and nerve [28]. Therefore, it is necessary to develop a 3D-printed all-natural polymer hydrogel scaffold with biocompatibility, biodegradability as well as high mechanical properties for cornea regeneration.

Ink plays a decisive role in the physico-chemical and biological properties of 3D printing hydrogel scaffold [29, 30]. As for digital photocuring processing (DLP) printing of natural polymer hydrogel, the mechanical properties of hydrogels are restricted by the low concentration of natural polymer ink and the high chemical crosslinking density-induced network brittleness after photocrosslinking. These limitations determine that natural polymer hydrogels printed by DLP are often weak and brittle [31–33]. While extrusion printing requires the ink to have a high viscosity, which is positively correlated to the strength of the printed hydrogel. However, printing a high-strength all-natural polymer hydrogel is still a challenge due to the inherent brittleness of the most natural polymers themselves as well as the sacrifice of mechanical strength from the 3D printing process. Temperature-sensitive inks based on hydrogen bonding interactions provide an ideal option for 3D printing of high-strength hydrogels, which can be extruded smoothly in the sol (or soft gel) state at a high temperature and eventually achieve high strength in the gel state after printing at a low temperature [34]. Recently, our team found that a blend of a low concentration of gelatin (Gel) and a low concentration of carbohydrazide-modified alginate (Alg-CDH) could coagulate into high-strength hydrogel (Gelatin/HAlg) after centrifugation due to the formation of hydrogen bonds between the hydrazide and gelatin [35]. In this gel network, the hydrazide structure of Alg-CDH could form high-density hydrogen-bonded cross-linking with the gelatin molecular chain, which served to simulate the regulatory effect of glycosaminoglycans on the orthogonal collagen fibers structure. However, this Gelatin/HAlg high-strength hydrogel based on the coacervation-centrifugation method cannot be used as a 3D printing ink.

**Figure 1.**
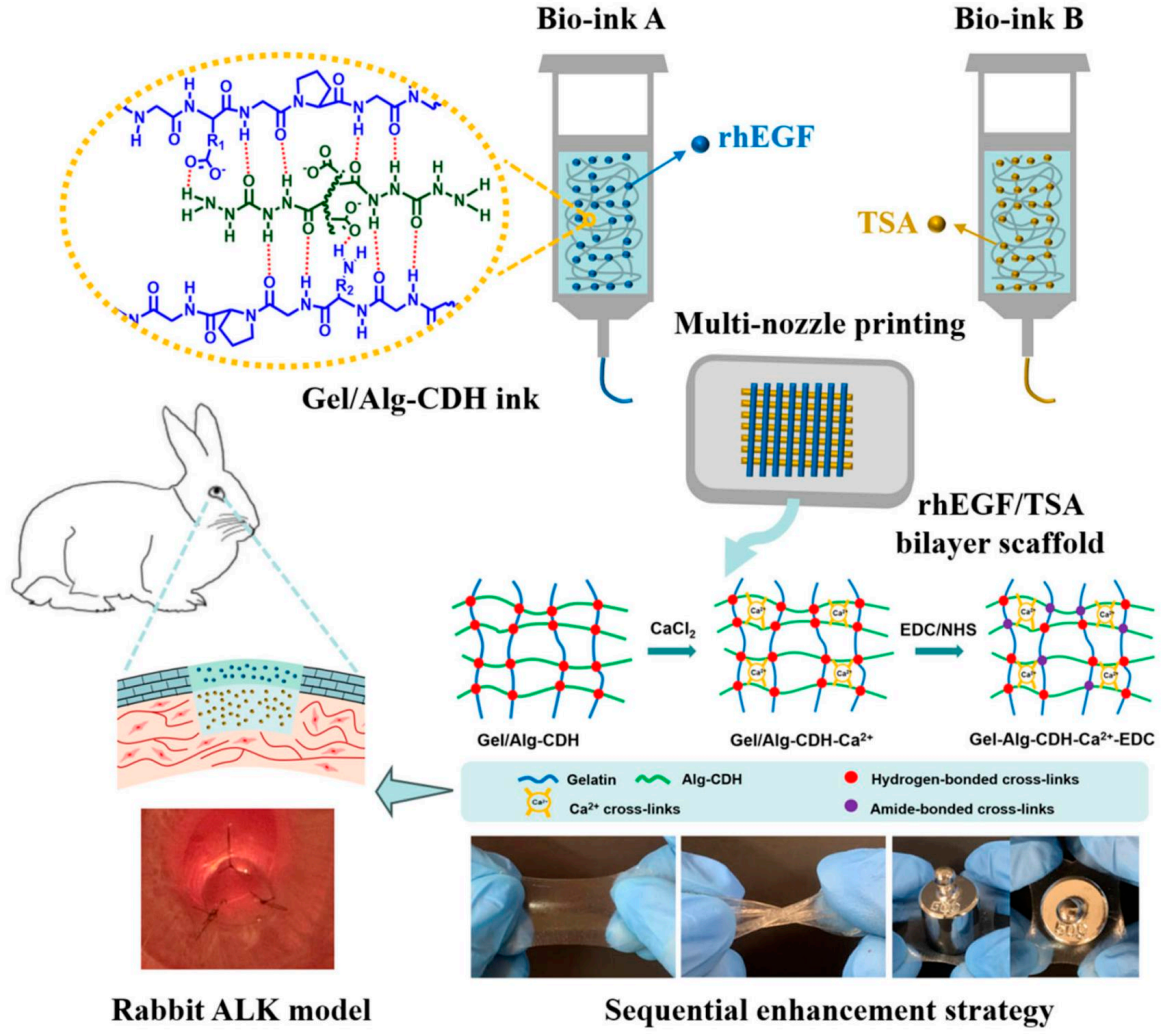
Schematic illustration of 3D-printed rhEGF/TSA hydrogel bilayer scaffolds using Gel/Alg-CDH inks, the sequential strengthening strategy, and application of the bilayer scaffold in rabbit corneal ALK models.

In this study, we developed an extrusion printing natural polymer ink (Gel/Alg-CDH) by blending a high concentration of Gel with a high concentration of Alg-CDH (Figure 1). This hydrogen bonding crosslinked Gel/Alg-CDH ink possessed a thermo-sensitivity and enhanced viscosity, making it very suitable for extrusion printing requirements. In addition, the printed Gel/Alg-CDH hydrogel was sequentially cross-linked by CaCl_2_ and 1-ethyl-3-(3-dimethylaminopropyl) carbodiimide hydrochloride (EDC)/N-hydroxysuccinimide (NHS) solution. The Ca^2+^ cross-linking Alg-CDH composition could further increase the physical cross-linking points, while low concentration of EDC/NHS treatment could catalyze the amide reaction of amino and carboxyl groups in Gel/Alg-CDH hydrogel to form moderate chemical crosslinking, which synergistically contributed to the considerable enhancement in the mechanical properties and physiological stability. Eventually, a strong physical crosslinking/moderate chemical crosslinking wholly natural polymer hydrogel with excellent mechanical properties and a J-shaped stress–strain curve similar to that of natural corneal tissue was obtained. The printed Ca^2+^-EDC/NHS crosslinked Gel/Alg-CDH (termed as Gel/Alg-CDH-Ca^2+^-EDC) hydrogel was highly good transparency, swelling-stable and biodegradable, suggesting its great potential as tissue engineering scaffold for cornea regeneration. More importantly, this tough wholly natural polymer hydrogel had a better suture retention capacity to withstand the surgical suture of anterior lamellar keratoplasty (ALK). In order to simulate the complex physiological structure of the cornea and meet the different requirements of the stromal and epithelial layer during corneal regeneration, a bilayer cornea scaffold loaded with recombinant human epidermal growth factor (rhEGF) on the top layer and Trichostatin A (TSA) on the bottom layer was constructed via a multi-nozzle printing system. *In vitro* experiments showed that rhEGF-loaded hydrogel layer could promote the proliferation and migration of rabbit cornea epithelial cells (rCECs), while TSA-loaded hydrogel layer could inhibit the α-SMA expression of TGF-β1-induced fibroblast fibrosis. Finally, we demonstrated that the rhEGF/TSA-loaded bilayer hydrogel scaffolds could promote cornea regeneration and inhibit scar tissue formation in a rabbit ALK model.

## Materials and methods

### Materials

Gelatin (Gel, Type A from porcine, 300 g bloom) was purchased from Sigma-Aldrich. Sodium alginate (Alg, low viscosity) was purchased from Alfa-Aesar. EDC and NHS were purchased from Beijing InnoChem Technology Co., Ltd. 1-hydroxybenzotrizole (HOBT, 98%) and carbohydrazide (CDH, 97%) were ordered from Heowns (Tianjin, China). Calcium chloride (CaCl_2_) was purchased from Aladdin (Shanghai, China). rhEGF and TSA were purchased from Sigma-Aldrich. All the other reagents were of analytical grade and used without further purification.

### Synthesis of Alg-CDH

Carbohydrazide-modified sodium alginate (Alg-CDH) was synthesized as described previously [35]. Firstly, 2.0 g alginate was added to 200 ml deionized water and dissolved at room temperature. Then 360 mg EDC, 540 mg HOBT and 360 mg CDH were successively added into the solution and reacted at room temperature for 48 h. Finally, the reacted solution was transferred to a dialysis bag (8000 Da), dialyzed in deionized water for 3 days and freeze-dried to obtain the Alg-CDH product.

### Preparation of Gel/Alg-CDH inks

Firstly, the binary phase diagram was investigated by mixing different concentrations of Gel solution and Alg-CDH solution with each other. And the preparation of Gel/Alg-CDH ink was achieved by simple blending of Gel solution and Alg-CDH solution. Here, the quality ratio of Gel and Alg-CDH is controlled at 10:1 with Gel as the main component, which is similar to the composition of the extracellular matrix. Specifically, gelatin was dissolved in deionized water at 45°C to obtain Gel solutions of 20 w/v%, 30 w/v% and 40 w/v%. Alg-CDH was dissolved in deionized water at 37°C to obtain Alg-CDH solutions of 2 w/v%, 3 w/v% and 4 w/v%. Then, 20 w/v% Gel and 2 w/v% Alg-CDH, 30 w/v% Gel and 3 w/v% Alg-CDH, 40 w/v% Gel and 4 w/v% Alg-CDH were mixed uniformly. The obtained inks were recorded as Gel/Alg-CDH-10-1.0, Gel/Alg-CDH-15-1.5 and Gel/Alg-CDH-20-2.0 (Gel/Alg-CDH-x-y, x is the mass volume fraction of Gel, y is the mass volume fraction of Alg-CDH), respectively. As a control, we prepared Gel/Alg inks (Gel/Alg-10-1.0, Gel/Alg-15-1.5 and Gel/Alg-20-2.0) by mixing Gel solution and Alg solution in the same method.

### Rheological characterization of Gel/Alg-CDH inks

The dynamic rheological behaviors of Gel/Alg-CDH inks were evaluated using a rheometer (MCR302, Anton Paar) with a temperature-controlled Peltier plate system. A 25-mm diameter parallel plate geometry and a working gap distance of 0.8 mm were selected for all tests. A strain of 1% was chosen for measuring other rheological behaviors under the oscillatory mode to make the hydrogel in the linear viscoelastic region. To evaluate the thermoresponsive behavior of Gel/Alg-CDH inks, the temperature ramp tests were conducted with a constant frequency at 1 Hz and a strain at 1% over a temperature range from 25°C to 60°C at 5°C min^−1^. To explore the shear-thinning behavior of Gel/Alg-CDH inks, the shear rate ramp tests (shear rate: 1–1000 1/s) were conducted with a constant frequency (1 Hz) at the gel–sol transition temperature of inks. In addition, in order to evaluate the self-recovery behaviors of Gel/Alg-CDH inks in response to the sharp temperature change, the cyclic temperature sweeps were performed at a constant frequency (1 Hz) and shear strain (1%). The alternative temperature was changed from a lower value (25°C) to a higher one (38°C for Gel/Alg-CDH-10-1.0; 40°C for Gel/Alg-CDH-15-1.5; 42°C for Gel/Alg-CDH-20-2.0) with a retention time of 250 s and an interval time of 150 s. Furthermore, the alternate step strain sweeps were also measured at a constant frequency (1 Hz) and 25°C. Amplitude oscillatory strains were switched from small strain (1%) to subsequent large strain (100%, 200% and 400%) with a 30-s interval between each strain stage. As a control, Gel/Alg inks were also subjected to the aforementioned rheological tests as the similar parameters.

### Printability of Gel/Alg-CDH inks

The printability of Gel/Alg-CDH ink was evaluated using a 3D bioprinter (Bio-Architect^®^ SR, Regenovo, China) with a low-temperature deposition platform. The desired 3D models designed from the CAD/CAM software or reconstructed from the Micro-CT data via Mimics Research 20.0 software (for rabbit’s cornea) were used for obtaining the corresponding G-code files through the CuraEngine slice software, which directed the layer-by-layer stack protocol to build up the designed architectures. Subsequently, the Gel/Alg-CDH inks were printed according to the following protocols. The Gel/Alg-CDH inks were loaded in a 5-ml cylinder and the temperature was controlled at 34°C for Gel/Alg-CDH-10-1.0, 35°C for Gel/Alg-CDH-15-1.5 and 36°C for Gel/Alg-CDH-20-2.0. After calibrating the platform and nozzle of the 3D bioprinter, a proper pressure (0.15–0.5 MPa) was applied to induce the flow of ink through a 0.34-mm needle (23G). The printed hydrogel structures were deposited on the platform at 20°C. Finally, the printed hydrogel structure was transferred to a 4°C refrigerator for a sequential strengthening strategy. To evaluate the printability of the Gel/Alg-CDH inks, orthogonal grids were printed and the printability index (Pr) was calculated by the following equation [36].

(1) $$\mathrm{Pr}=\frac{L^{2}}{16A},$$

where *L* is the perimeter and *A* is the area of the grid.

### The sequential strengthening strategy of Gel/Alg-CDH hydrogels

In order to improve the mechanical properties of the hydrogel, we performed a sequential enhancement treatment on the printed hydrogel. First, the printed Gel/Alg-CDH hydrogel was immersed in 50 mM CaCl_2_ solution for 10 min to achieve Ca^2+^ cross-linking of the alginate composition, and the treated hydrogel was recorded as Gel/Alg-CDH-Ca^2+^. Then, Gel/Alg-CDH-Ca^2+^ was washed with deionized water and immersed in 10 mM EDC/NHS solution for 1, 3, 5 and 10 min, respectively, to achieve varying degrees of chemical crosslinking. Finally, the physical–chemical synergistic hydrogel treated with the sequential strengthening strategy was recorded as Gel-Alg-CDH-Ca^2+^-EDC.

### Determination of crosslinking density and amide reaction efficiency

The structural parameter crosslinking densities (*ν*_c_) of Gel-Alg-CDH-20-2.0-Ca^2+^-EDC hydrogels with different EDC/NHS treatment times (0, 1, 3, 5 and 10 min) were calculated by rheological equation [34]:

(2) $$\nu_{c}=\frac{\;{G\prime }_{p}N_{A}}{RT},$$

where the *N*_A_, *R* and *T* represent the Avogadro constant (6.022 × 10^23^ mol^−1^), the universal gas constant (8.314 J (mol K ^−1^) and the thermodynamic temperature (K), respectively. And the frequency sweep tests (0.1–10 Hz) were also conducted at room temperature to obtain the rubber-elastic plateau *G*′_p_ (Pa).

The amide reaction efficiencies (*ξ*) of Gel-Alg-CDH-20-2.0-Ca^2+^-EDC hydrogels with different EDC/NHS treatment times (0, 1, 3, 5 and 10 min) were calculated using ninhydrin colorimetry. First, 300 mg Gel-Alg-CDH-20-2.0-Ca^2+^-EDC hydrogels with different EDC/NHS treatment times were ground and crushed, then added into a 5 ml centrifuge tube. About 1.5 ml PBS buffer (pH = 6.5) and 1 ml Ninhydrin solution (2 w/v %) were sequentially added to the centrifuge tube. All centrifuge tubes were incubated at 100°C for 15 min, and the OD_570 nm_ value of the cooled supernatants was measured using a microplate reader (Infinite M200 PRO, Tecan). The reaction efficiencies (*ξ*) were calculated by the following equation:

(3) $$\xi (\%)=(1-\frac{\;A_{t}-A_{c}}{A_{0}-A_{c}})\times 100\%,$$

where *A*_t_ is the OD_570 nm_ of hydrogels with different EDC/NHS treatment times (1, 3, 5 and 10 min), *A*_0_ is the OD_570 nm_ of Gel/Alg-CDH-20-2.0-Ca^2+^ hydrogels (0 min) and *A*_c_ is the OD_570 nm_ of the control group.

### Density functional theory study

To study the molecular configurations and interaction strengths among Gel/Alg and Gel/Alg-CDH, we calculated the interaction energies based on density functional theory (DFT) as described in the literature [37]. Herein, the gelatin molecule is simplified to the representative sequence hydroxyproline-proline-glycine (Hyp-Pro-Gly), and the alginate molecule is simplified to monosaccharide units. The DFT program DMol3 in Material Studio (Accelrys, San Diego, CA) was used to perform all simulations.

### Physicochemical characterization of hydrogels

#### Measurement of mechanical performances

The mechanical performances of all hydrogels were measured using an Instron 2344 Microtester at room temperature. In the tensile tests, the 3D-printed hydrogel and the printed hydrogel with different crosslinking treatments were cut into dumbbell-shaped samples according to the ASTM standard and the thickness and length of samples were measured before tests. The stretching rate was set to 50 mm min^−1^. The tensile strength, elongation at break and toughness were obtained directly from the stress–strain curves and tensile modulus were determined by the slope of the initial linear region (10–20% strain) of the stress–strain curves. Besides, in order to evaluate the mechanical properties in a physiological environment, Gel-Alg-CDH-20-2.0-Ca^2+^-EDC hydrogels were immersed in PBS at 37°C for 3 and 7 days for tensile testing. In addition, we printed the Gel-Alg-CDH-20-2.0 hydrogel cylinder with a height of 2 mm, immersed it in CaCl_2_ solution for 30 min and EDC/NHS solution for 30 min successively, and then tested its compression modulus (10–20% strain). The compressive rate was set to 10 mm min^−1^.

#### Evaluation of optical properties

The light transmittance of hydrogel was measured with a UV-vis spectrophotometer (GENESYS 180, ThermoFisher Scientific) over a wavelength range from 400 to 750 nm using PBS as a blank at room temperature. All samples were cut into long strips and measured in the quartz dish.

#### Swelling behavior of hydrogels

The hydrogel samples were immersed in PBS at 37°C and weighed at the predetermined time to monitor the increased weight. The swelling degree (SD) of hydrogel was calculated as the following equation:

(4) $$\mathrm{SD}\;(\%)=\frac{W_{t}-W_{0}}{W_{0}}\times 100\%,$$

where *W*_t_ was the sample weight at the predetermined time points and *W*_0_ was the initial weight.

#### Enzymatic degradation assay

The lyophilized hydrogels were recorded with its initial mass m_0_ and then incubated in the PBS solution of 0.1 U/ml collagenase A (Sigma) at 37°C. The solution was removed from the centrifuge tubes at predetermined time points and the remaining mass *m*_t_ of the hydrogel samples at each moment was recorded after freeze-drying. The percentage of the remaining mass of the hydrogel was calculated by the following equation:

(5) $$\mathrm{Remaining}\;\mathrm{mass}\;(\%)=\frac{m_{t}}{m_{0}}\times 100\%$$

#### Suture retention capacity

Regular rectangle samples were prepared for suture retention capacity tests [38]. In brief, one end of the hydrogel sample (about 500 μm) was directly clamped on the Instron 2344 Microtester, and the other end was passed through by a 10-0 ophthalmic suture. The puncture point of the suture is 5 mm away from the edge of the sample, and then the suture was also clamped on the microtester. Perform tensile testing at a speed of 50 mm min^−1^ and record the load–displacement curve during the testing process.

### Preparation of rhEGF/TSA bilayer hydrogel scaffold

In order to simulate the physiological structure of the epithelial stromal of corneas, we printed a rhEGF/TSA bilayer hydrogel scaffold using two types of Gel/Alg-CDH inks. Firstly, rhEGF and TSA were added to Gel/Alg-CDH-20-2.0 ink to prepare ink A (final rhEGF concentration: 2 μg/ml) and ink B (final TSA concentration: 5 μmol/l), respectively. Then, the rhEGF/TSA bilayer hydrogel scaffold was obtained by printing layer by layer in the bio-printer through a multi-nozzle printing system using ink A and ink B. We also printed rhEGF-loaded Gel/Alg-CDH-20-2.0 and TSA-loaded Gel/Alg-CDH-20-2.0 for the release assay of rhEGF and TSA and *in vitro* cell experiments. These hydrogel scaffolds were also treated with the sequential enhancement treatment as in ‘The sequential strengthening strategy of Gel/Alg-CDH hydrogels’ section.

rhEGF release assay: Briefly, 0.2 g rhEGF-loaded (2 μg/ml) Gel-Alg-CDH-20-2.0-Ca^2+^-EDC hydrogel was immersed in 1 ml PBS and incubated at 37°C in a 5-ml centrifuge tube. Supernatants were removed and resupplied at 0, 2, 4, 6, 8, 12, 24 and 36 h. The rhEGF concentration of samples at each time point was determined with the Human EGF ELISA Kit (Boster).

TSA release assay: Briefly, 1 g TSA-loaded (50 μmol/l) Gel-Alg-CDH-20-2.0-Ca^2+^-EDC hydrogel was immersed in 3 ml PBS and incubated at 37°C in a 5-ml centrifuge tube. The released TSA concentration of samples at each time point was measured at a relevant UV wavelength (365 nm) according to the measured standard curve of TSA concentration.

### Cytocompatibility of hydrogels in vitro

#### Cell culture

rCECs (BFB Company, China), rabbit adipose-derived mesenchymal stem cells (rASCs, Cyagen Company, China) and mouse fibroblast cells (L929, BFB Company, China) were used to verify the cytocompatibility of hydrogels *in vitro*. rCECs and L929 were cultured in Dulbecco’s modified eagle media with 10% fetal bovine serum albumin and 1% penicillin/streptomycin. rASCs were cultured in complete media provided by Cyagen company. All cultures were incubated at 37°C in a humidified atmosphere of 5% CO_2_ in the cell incubator. Subculture by trypsinization with trypsin/EDTA solution was performed when the cells reached to 80% confluence. Cells at passages 3–7 were used for the experiments. The hydrogel used in cell experiments refers to Gel-Alg-CDH-20-2.0-Ca^2+^-EDC hydrogel.

#### Cell adhesion assay

To evaluate the adhesion ability of cells cultured on Gel-Alg-CDH-20-2.0-Ca^2+^-EDC hydrogel, 1 × 10^4^ rASCs cells were seeded on the sterilized hydrogel (a diameter of 10 mm) in a 48-well plate and incubated for 1, 3 and 5 days. At each time point, Live/Dead staining with Calcein AM/PI kit was conducted following the manufacturer’s protocol (MCE), and live cells (green)/dead cells (red) were imaged using a fluorescence microscope (EVOS M5000) on days 1, 3 and 5. The CCK-8 assay was used to analyze cell proliferation on the surface of hydrogels. About 30 μl CCK-8 and 300 μl complete media were added to each well and the cells were incubated in the dark for 3 h at 37°C. After incubation, the absorbance at 450 nm of each well was immediately read.

#### Cell proliferation assay

To evaluate the promoting effect of rhEGF-loaded hydrogel scaffold on cornea epithelial cell proliferation, Cell proliferation of rCEC treated with Gel-Alg-CDH-20-2.0-Ca^2+^-EDC hydrogel and rhEGF-loaded hydrogel were calculated by CCK-8 assay. Firstly, 8 × 10^4^ rCECs cells were seeded into a 48-well plate containing 300 μl serum-free DMEM in each well. Then, the hydrogel and rhEGF-loaded hydrogel (a diameter of 5 mm) were added into each well, respectively. After incubation for 12 and 24 h, Live/Dead staining and CCK8 assay were conducted.

#### Cell scratch assay

To determine the effect of rhEGF-loaded hydrogel scaffold on cornea epithelial cell renewal, the migration capacity of rCECs treated with Gel-Alg-CDH-20-2.0-Ca^2+^-EDC hydrogel and rhEGF-loaded hydrogel was evaluated. Firstly, 1 × 10^5^ rCECs in 500 μl complete medium were seeded on a 24-well plate and incubated until 100% confluency. A scratch was made by a sterilized 200 μl pipette tip and cleaned with PBS, and then 500 μl serum-free DMEM culture medium was added into the 48-well plate. Finally, the sterilized hydrogel and rhEGF-loaded hydrogel (a diameter of 5 mm) were added. After 12 and 24 h, the cells were imaged using a biomicroscope and the relative cell migration distance within the scratched area was analyzed.

#### Biocompatibility of TSA-hydrogels

We evaluated the cytotoxicity of TSA-loaded hydrogels to rASCs and the inhibition of α-SMA protein expression of TGF-β1-mediated L929 fibrosis. Firstly, 1 × 10^4^ rASCs in 200 μl complete medium were seeded on a 96-well plate and incubated for overnight. Then, the hydrogel and TSA-loaded hydrogel (a diameter of 5 mm) were added into the 96-well plate. rASCs of all groups were incubated for 3 days, and CCK-8 assay and live/dead staining were performed on day 1, day 2 and day 3 to evaluate the effect of TSA on cell activity.

The effect of released TSA on TGF-β1-mediated myofibroblast generation was assessed by α-SMA/DAPI immunofluorescence staining. Firstly, 5 × 10^5^ L929 in 1 ml complete medium was seeded on a 6-well plate containing cell crawl sheets and incubated for 24 h. Hydrogels and TSA-loaded hydrogels (100 mg/ml) were immersed in serum-free DMEM medium for 24 h to obtain the extracts, and then TGF-β1 (5 ng/ml) was added to the extracts. About 1 ml of two extracts was added to the 6-well plate instead of the original complete medium in each well, and the cells continued to be incubated for 24 h. Finally, cell crawls were stained with α-SMA/DAPI immunofluorescence as described in the literature [39].

### *In vivo* assessment of hydrogel in a rabbit anterior lamellar keratoplasty

#### Surgical procedures

All the animal experiments were carried out according to the guidelines of the Tianjin Medical Experimental Animal Care, and animal protocols were approved by the Institutional Animal Care and Use Committee of Yi Shengyuan Gene Technology (Tianjin) Co., Ltd (no. YSY-DWLL-2023190).

New Zealand white rabbits (male, 12–14 weeks old, 2.5–3 kg) were randomly divided into three groups (six rabbits per group), including the rhEGF/TSA bilayer hydrogel group, the hydrogel group and the defect group (control group). All surgeries were performed on the left eyes of rabbits, and the right eyes were served as the normal group. Inject thiazide hydrochloride (0.1–0.2 ml) into the thigh muscles of rabbits for anesthesia before surgery. ALK is performed under a surgical microscope (Carl Zeiss f170), using a customized 3 mm trephine and 15° knife to remove the cornea epithelium and a certain depth of stroma (∼150 μm). Then, the rhEGF/TSA bilayer hydrogel (about 300 μm, rhEGF layer facing upwards) and hydrogel scaffolds were punched with the 3 mm trephine and carefully placed on the cornea defect area, respectively. These hydrogel scaffolds have sufficient mechanical properties, so we directly suture these hydrogel scaffolds on the surrounding cornea tissue with 10-0 ophthalmic sutures. The control group only removed the anterior lamellar layer. Postoperative management includes daily dropping of levofloxacin hydrochloride solution for a week to prevent infection and maintain moisture. The suture was removed on day 7 after surgery.

#### Slit-lamp and anterior segment optical coherence tomography evaluation

Slit lamp (RTvue XR, OPTOVUE) was used to monitor the anterior ocular tissue after keratoplasty, and on day 7 and day 14 post-operation. At the same time, cross-sectional images of cornea tissue were obtained by anterior segment optical coherence tomography (AS-OCT) (Haag-Streit BX900) to evaluate the cornea repairment.

#### Histological assessment

On the 14th day after surgery, all groups of rabbits were sacrificed and the eyeballs were excised and fixed with 4% (v/v) paraformaldehyde, followed by embedding the cornea with paraffin. The paraffin sections (5 μm) were stained with hematoxylin and eosin (H&E) and were viewed under a microscope for histological analysis. In order to further analyze the cornea repair effect of each group, immunofluorescence staining of cytokeratin 3 (CK3), collagen type I (Col I), lumican (LUM) and alpha smooth muscle actin (α-SMA) were performed on the sections, as described in the literature [23].

### Statistical analysis

Data were expressed as means ± standard deviations (*n* ≥ 3). SPSS 27.0 was used for statistical analysis of data. A value of *P* < 0.05 was considered statistically significant, and ns, *, **, *** represent *P* > 0.05, *P* < 0.05, *P* < 0.01, *P* < 0.001, respectively.

## Results and discussion

### Preparation of Gel/Alg-CDH inks

Alg-CDH was synthesized by coupling the carboxyl group of Alg with the amino group of carbohydrazide under EDC/NHS catalysis as reported previously [35], and the optimal feed ratio corresponding to the maximum degree of substitution was selected to couple more hydrazide moieties to Alg. As described in our previous work, dilute gelatin aqueous solution (3 w/v%) and dilute Alg-CDH aqueous solution (0.3 w/v%) were mixed and allowed to stand for a few minutes to produce white flocculent hydrogel due to the strong physical interaction between gelatin and Alg-CDH. Then, irreversible and strong Gelatin/HAlg hydrogel was obtained by high-speed centrifugation to remove supernatant (Supplementary Figure S1A). However, this Gelatin/HAlg hydrogel was thermally irreversible. As shown in Supplementary Figure S1B, with the increase of temperature, the Gelatin/HAlg hydrogel always maintained a higher storage modulus *G*′ (> *G*″). Even at a high temperature of 80°C, sol-gel transformation did not occur. And the high strength leads to great resistance in the injection process of the hydrogel in the small aperture needle. Therefore, this Gelatin/HAlg hydrogel was not suitable for use as an ink for extrusion 3D printing.

In this study, we investigated the blends of high-concentration gelatin solution and high-concentration Alg-CDH solution. As shown in Supplementary Figure S2, the blends gradually became homogeneous and transparent with increasing gelatin concentration and Alg-CDH concentration. Three different states of the blends were formed: (I) precipitation, (II) opaque homogeneous mixture and (III) transparent homogeneous mixture. Among them, the precipitate in region I can be compressed by centrifugation to obtain a high-strength hydrogel, which cannot be used as ink, and the homogeneous blends in regions II and III are temperature-sensitive and can be used as extrusion printing ink. Here, we focus on the representative mixtures Gel/Alg-CDH-10-1.0, Gel/Alg-CDH-15-1.5 and Gel/Alg-CDH-20-2.0 from regions II and III (recorded as Gel/Alg-CDH-x-y, x is the mass volume fraction of Gel, y is the mass volume fraction of Alg-CDH). The maximum solid content of the representative mixtures (Gel/Alg-CDH-20-2.0) was close to that of natural corneal tissue (∼20%). As shown in Supplementary Figure S3, although hydrogen bonding interactions still existed between the high-concentration gelatin solution and high concentration Alg-CDH solution, the macromolecular chains were confined to the concentrated polymer network with high viscosity and could not form precipitates; as a result, Gel/Alg-CDH-10-1.0, Gel/Alg-CDH-15-1.5 and Gel/Alg-CDH-20- 2.0 formed a homogeneous viscous polymer solution after mixing at 45°C and gradually turned into hydrogels with decreasing temperature. Gel/Alg-CDH-10-1.0 was whitish, possibly due to the formation of aggregates, and this phenomenon was consistent with the formation of the precipitate in region I. These results demonstrated the potential of concentrated Gel/Alg-CDH as an extruded 3D-printing ink.

### Rheological properties of Gel/Alg-CDH inks

**Figure 2.**
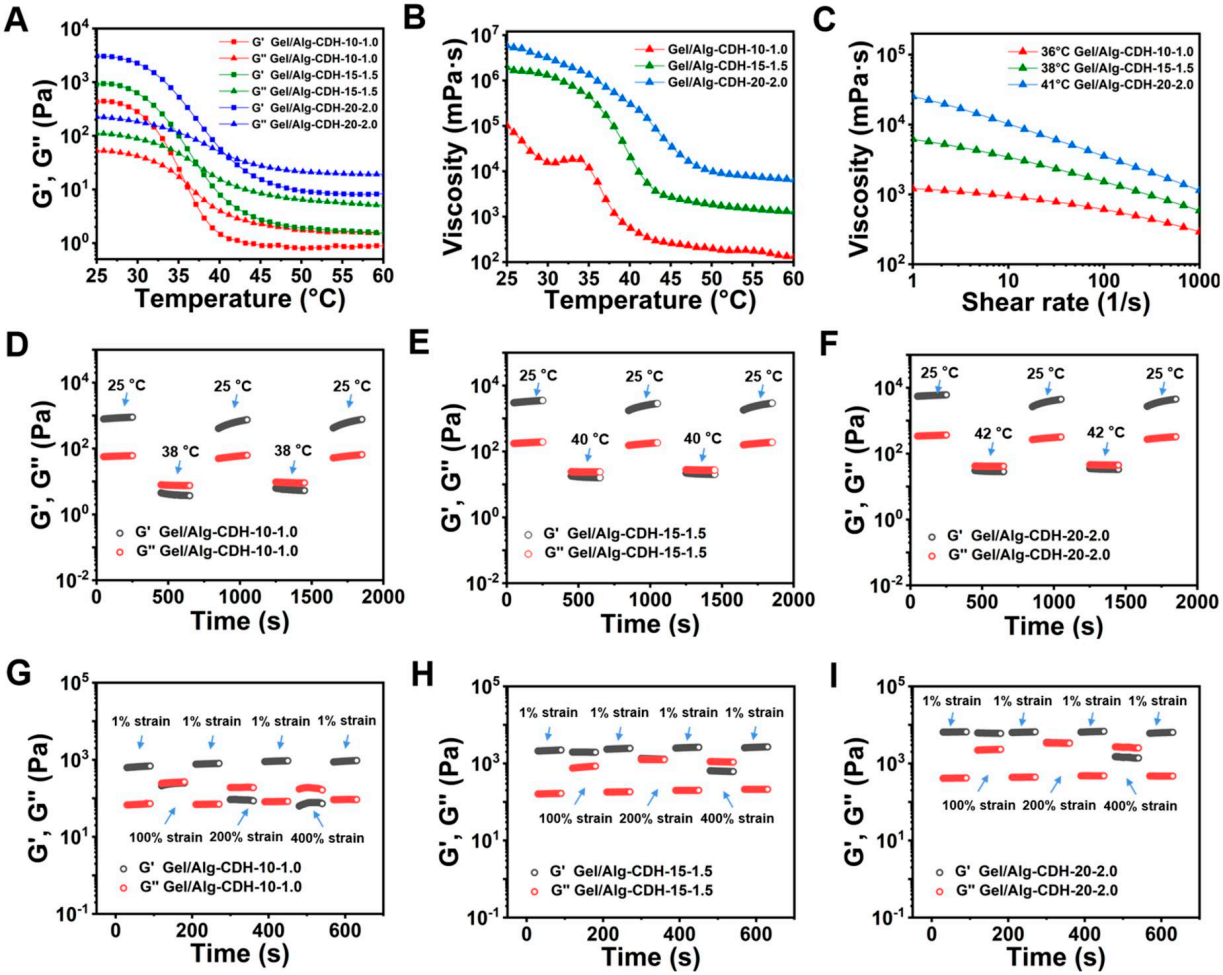
Rheological properties of Gel/Alg-CDH inks. (**A**) Variation of storage modulus (*G*′) and loss modulus (*G*″) and (**B**) viscosity of Gel/Alg-CDH inks as a function of temperature. (**C**) Shear-thinning behavior of Gel/Alg-CDH inks at their gel–sol transition temperatures. (**D–F**) Cyclic temperature sweep and (**G–I**) cyclic strain sweep of Gel/Alg-CDH inks (D, G: Gel/Alg-CDH-10-1.0; E, H: Gel/Alg-CDH-15-1.5; F, I: Gel/Alg-CDH-20-2.0).

In order to evaluate their extrusion printability and select the optimal printing ink system, the key rheological properties of Gel/Alg-CDH were systematically investigated (e.g. gel–sol transition, viscosity–temperature curve, shear-thinning characteristic, self-recovery). As a contrast, we also tested the rheological properties of the unmodified Gel/Alg. The temperature-dependent gel–sol transition of prepared Gel/Alg-CDH was studied by temperature amplitude scanning test. As illustrated in Figure 2A, in the low-temperature stage, the storage modulus (*G*′) of Gel/Alg-CDH was greater than the loss modulus (*G*″), and these two values gradually decreased with increasing temperature. At higher temperatures, *G*′ and *G*″ started to cross, and *G*″ was gradually dominant over *G*′, suggesting that Gel/Alg-CDH underwent a gel–sol transition due to the dissociation of hydrogen bonding [40, 41]. The gel–sol transition temperatures of Gel/Alg-CDH-10-1.0, Gel/Alg-CDH-15-1.5 and Gel/Alg-CDH-20-2.0 were determined as 36°C, 38°C and 41°C, respectively. In comparison, Gel/Alg also exhibited the gel–sol transition behavior with temperature increasing due to the thermosensitivity of gelatin components (Supplementary Figure S4A). However, the gel–sol transition temperature of Ge/Alg-10-1.0, Gel/Alg-15-1.5 and Gel/Alg-20-2.0 was 32°C, 34°C and 35°C, respectively, which were lower than that of the corresponding Gel/Alg-CDHs. In addition, at the same temperature, *G*′ and *G*″ of Gel/Alg was also much lower than that of the corresponding Gel/Alg-CDH, and this trend was particularly significant at high temperatures (higher than the gel–sol transition temperature). These results demonstrated that the introduction of carbohydrazide in Alg-CDH significantly enhanced the physical interaction between Gel and Alg-CDH. This strong physical interaction was mainly originated from the strong hydrogen bonding interactions between the carbohydrazide structure and the gelatin molecular chain, as well as the electrostatic interaction between the positive charge of gelatin and the negative charge of Alg-CDH. Therefore, Gel/Alg-CDH could form strong hydrogel at low temperatures and maintain high cohesion at high temperatures. The viscosity of inks was also an important evaluation indicator of printability. Next, we also investigated the temperature-dependent viscosity curve of Gel/Alg-CDH. As shown in Figure 2B, the viscosity of Gel/Alg-CDH-20-2.0 and Gel/Alg-CDH-15-1.5 gradually decreased with the increase of temperature, and the curve slope became higher upon approaching the gel–sol transition temperature. Although Gel/Alg-CDH-10-1.0 also showed the overall trend of viscosity decreasing with the increase in temperature, there was a small platform near the gel–sol transition temperature, which might be related to the aggregates formed in the Gel/Alg-CDH-10-1.0. Similarly, the viscosities of the three proportions of Gel/Alg (Supplementary Figure S4B) were lower than the viscosity of the corresponding Gel/Alg-CDH at the same temperature. The viscosity of Gel/Alg dropped very quickly and was very low at higher temperatures, which was not conducive to extrusion printing (higher viscosity is required to improve shape fidelity) [42]. At high temperatures, Gel/Alg-CDH-20-2.0 and Gel/Alg-CDH-15-1.5 had higher viscosities (near the gel–sol transition temperature), which were suitable for printing. In the following experiment, the shear-thinning characteristic of Gel/Alg-CDH that is also a vital prerequisite for extrusion printing was investigated. As shown in Figure 2C, Gel/Alg-CDH-20-2.0, Gel/Alg-CDH-15-1.5 and Gel/Alg-CDH-10-1.0 all showed significant shear-thinning behavior at their gel–sol transition temperature with the viscosities decreasing rapidly when the shear rate was increased. However, the viscosities of Gel/Alg-20-2.0, Gel/Alg-15-1.5 and Gel/Alg-10-1.0 changed slowly at the gel–sol transition temperature, and there was no obvious shear-thinning behavior (Supplementary Figure S4C). The reason for this phenomenon may be that Gel/Alg had a low cohesion and viscosity near the gel–sol transition temperature, so it was not sensitive to shear rate. This result once again proved the rheological superiority of Gel/Alg-CDH over Gel/Alg as the extrusion 3D-printing ink.

The extrusion printing of temperature-sensitive inks is usually achieved through a high-temperature/high-strain extrusion and low-temperature/low-strain deposition mode. Therefore, the alternate temperature sweep and the alternate strain sweep measurements were conducted to evaluate the thermosensitiveness and strain-induced recovery behavior of Gel/Alg-CDH. When subjected to the low temperature (25°C)–high temperature (38°C for Gel/Alg-CDH-10-1.0; 40°C for Gel/Alg-CDH-15-1.5; and 42°C for Gel/Alg-CDH-20-2.0) alternate treatment, the Gel/Alg-CDH showed a reversible gel–sol transition behavior and their mechanical properties could be rapidly recovered in response to temperature variations (Figure 2D–F). This result suggested that the temperature-dependent reversible hydrogen-bond reforming ability of Gel/Alg-CDH ink allowed for the completion of the extrusion-deposition process. Although Gel/Alg also exhibited a thermo-reversible gel–sol transition in the alternate temperature sweep measurements (Gel/Alg-10-1.0: 25°C–34°C; Gel/Alg-15-1.5: 25°C–36°C; Gel/Alg-15-1.5: 25°C–38°C), the *G*′ and *G*″ values of Gel/Alg are relatively close at low temperatures, and *G*′ is far lower than the corresponding *G*′ of Ge/Alg-CDH (Supplementary Figure S4D–F). This suggested that the hydrogen bonding strengthened Gel/Alg-CDH contributed to restorable mechanical properties and better printing fidelity. The alternate step strain sweep measurements were also performed to examine the strain-induced gel–sol transition of Gel/Alg-CDH. As displayed in Figure 2G–I, at a constant frequency (1 Hz) and temperature (25°C), Gel/Alg-CDH maintained a gel-like viscoelasticity at a low shear strain of 1%. In this case, *G*′ was greater than *G*″. As the shear strain changed from 1% to the higher value (100%, 200% or 400%), *G*″ gradually approached *G*′‘ (100% for Gel/Alg-CDH-10-1.0, 200% for Gel/Alg-CDH-15-1.5 and Gel/Alg-CDH-20-2.0), and finally surpasses *G*’ (*G*″ > *G*′, 200% for Gel/Alg-CDH-10-1.0, 400% for Gel/Alg-CDH-15-1.5 and Gel/Alg-CDH-20-2.0), implying the dissociation of hydrogen bonding cross-linking network of Gel/Alg-CDH at high shear strains. Gel/Alg-CDH-10-1.0 underwent the gel–sol transition at a lower strain than Gel/Alg-CDH-15-1.5 and Gel/Alg-CDH-20-2.0, because of the lower hydrogen bonding crosslinking density. When the shear strain came back to 1%, the shear modulus *G*″ and *G*′ of Gel/Alg-CDHs could immediately return to the initial values, indicating the rapid reconstruction of hydrogen bonding crosslinking. Although Gel/Algs also exhibited a shear strain-induced reversible gel–sol transition in alternate strain sweep measurements (Supplementary Figure S4G–I), Gel/Alg-20-2.0 was less reversible than Gel/Alg-CDH-20-2.0, with its *G*′ (∼600 Pa) at a low shear strain of 1% being much lower than the initial value (∼3300 Pa) after strain sweep. This mirrored the worse reversibility of the weak hydrogen bonding crosslinking network of Gel/Alg-20-2.0, while the strong hydrogen-bonded cross-link of Gel/Alg-CDH-20-2.0 led to better recovery of deformation. All these results showed that Gel/Alg-CDH possessed the most favorable rheological properties (including thermosensitive gel–sol transition, hydrogen-bonding enhanced viscosity, shear thinning, thermosensitive mechanical reversibility and shear strain-induced mechanical reversibility) for extrusion printing. These properties conferred excellent printability and print fidelity to Gel/Alg-CDH.

### Printability evaluation of Gel/Alg-CDH inks

**Figure 3.**
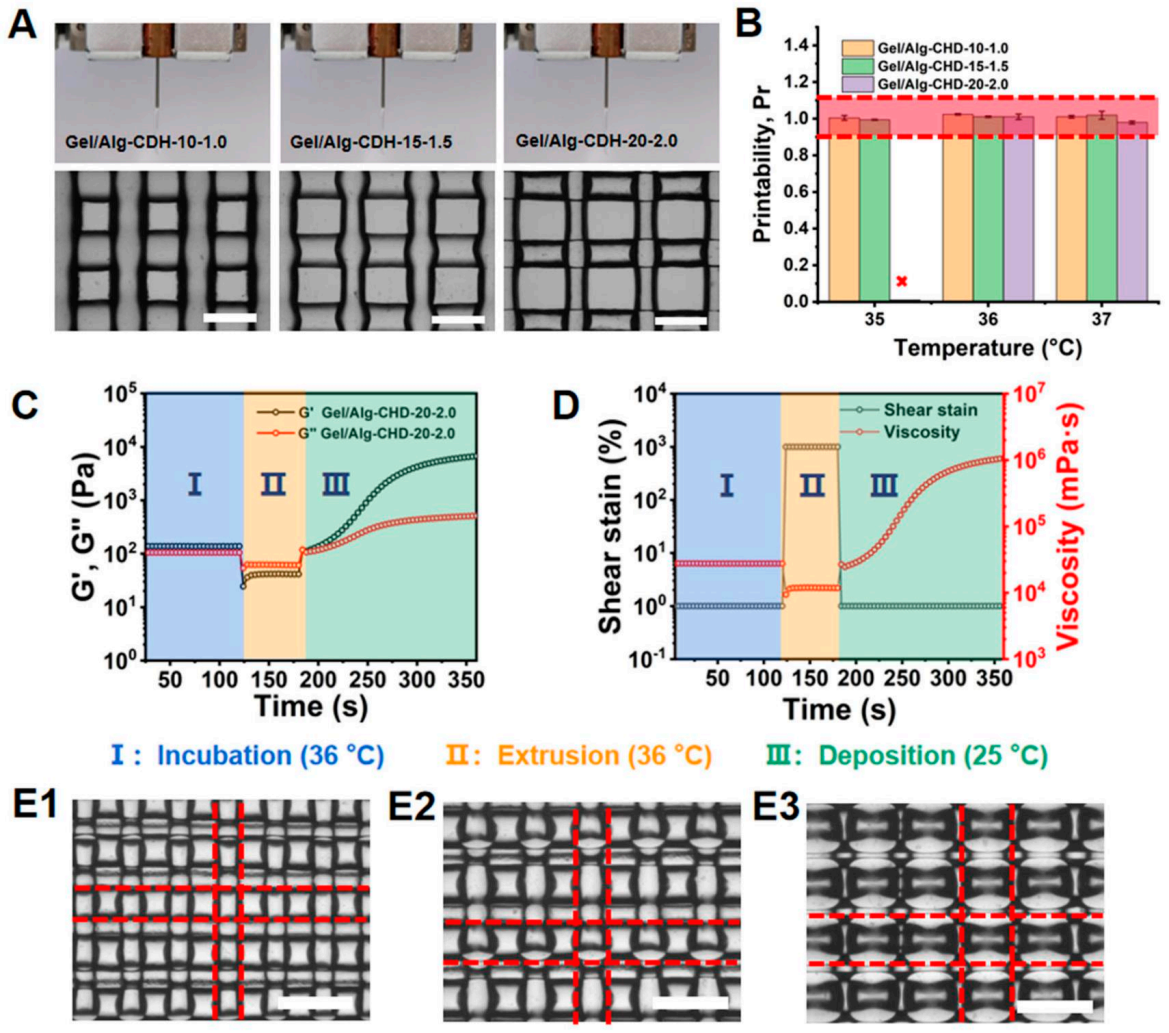
Printability of Gel/Alg-CDH inks. (**A**) Extruded filaments of Gel/Alg-CDH inks under optimized printing parameters and microscopic photographs of printed grid structure with Gel/Alg-CDH inks. Scale bars: 750 μm. (**B**) Semi-quantified Pr values of 3D-printed grid structure using Gel/Alg-CDH inks at different temperatures. (**C, D**) Variation of shear modulus (*G*′ and *G*″), shear strain and viscosity of Gel/Alg-CDH-20-2.0 ink in the rheological simulation of the extrusion printing process. (**E**) Printed orthogonal grids with different filament diameters and different spacings using Gel/Alg-CDH-20-2.0 ink under different air pressure and model path spacing (E1: 0.3 MPa, 0.5 mm; E2: 0.3 MPa, 0.6 mm; E3: 0.4 MPa, 0.7 mm). Scale bars: 750 μm.

We next directly evaluated the printability of Gel/Alg-CDH inks on the bio-printer. The appropriate ink temperature is crucial for the extrusion printing effect. If the temperature is near or higher than the gel–sol transition temperature, the ink is in an under-gel state (initial gel with a very low modulus) or solution state. Under this condition, the ink does not have enough viscosity to support the continuous extrusion of filaments, so the low modulus of extruded ink may cause the deposited structure to collapse easily and lose printing accuracy (Supplementary Figure S5A). Conversely, if the temperature is well below the gel–sol transition temperature, the ink is in a full-gel state, and extruded filaments may be broken during printing, resulting in the formation of irregular filaments and twisted meshes (Supplementary Figure S5B). Taking these factors into consideration, we finally chose to perform 3D printing at a barrel temperature slightly below the gel–sol transition temperature (34°C for Gel/Alg-CDH-10-1.0, 35°C for Gel/Alg-CDH-15-1.5 and 36°C for Gel/Alg-CDH-20-2.0). A platform temperature of 20°C was chosen to achieve rapid deposition solidification while maintaining connectivity between filaments, and the nozzle size was chosen as 23G for printing. Air pressure and nozzle movement speed were selected on merit. Under these conditions, Gel/Alg-CDH-10-1.0, Gel/Alg-CDH-15-1.5 and Gel/Alg-CDH-20-2.0 all exhibited good printability, with regular and well-defined printed orthogonal meshes and no significant collapse, as illustrated in Figure 3A and Supplementary Movie S1. Here, the printability index (Pr) was used to semi-quantitatively evaluate the printability of Gel/Alg-CDH inks near the body temperature for application as bio-inks. The closer the Pr of the ink was to 1, the higher its printing accuracy. As shown in Figure 3B, Gel/Alg-CDH-10-1.0 and Gel/Alg-CDH-15-1.5 had Pr values very close to 1 at 35°C, 36°C and 37° C. Gel/Alg-CDH-20-2.0 exhibited a good printability at 36°C and 37°C, but could not be continuously extruded smoothly at 35°C due to its excessive hydrogen bonding interaction at relatively low temperatures. This suggested that all Gel/Alg-CDH held potential as bio-inks. We further simulated the printing process of the Gel/Alg-CDH inks by rheological characterization. The 3D printing process was divided into three stages: incubation, extrusion and deposition. As shown in Figure 3C and D, during the incubation phase in the barrel, the Gel/Alg-CDH-20-2.0 ink was in a high temperature and low strain environment, so it was in a soft gel state and its low shear modulus (*G*′ of about 138 Pa) facilitated continuous extrusion while maintaining a high viscosity (about 2.7 × 10^4^ mPa⋅s) to prevent ink leakage from the nozzle. In the extrusion phase, when air pressure was applied to the incubated ink, gel–sol transition occurred to Gel/Alg-CDH-20-2.0 at a high temperature and high shear strain, so the shear modulus (*G*′ of about 42 Pa) and viscosity (about 1.2 × 10^4^ mPa⋅s) of the ink decreased sharply. This shear thinning allowed the ink to be smoothly extruded through the nozzle. During the deposition phase, with the high shear strain being released, the ink deposited on the low-temperature platform, and the hydrogen bonding interactions of Gel/Alg-CDH-20-2.0 disrupted at the high temperature and high shear strain was re-established. The ink rapidly returned to the gel state, and its shear modulus (*G*′ of about 6700 Pa) and viscosity (about 1 × 10^6^ mPa⋅s) rose to a high value. The rapid recovery of mechanical properties ensured the shape fidelity of Gel/Alg-CDH-20-2.0. The Gel/Alg-CDH-15-1.5 and Gel/Alg-CDH-10-1.0 also exhibited gel–sol–gel transition during the incubation softening—high-pressure extrusion—low temperature deposition process (Supplementary Figure S6). This process was accompanied by the partial dissociation–dissociation–reconstruction of the hydrogen-bonded crosslinking network of Gel/Alg-CDH. Notably, the modulus and viscosity of the deposited Gel/Alg-CDH-20-2.0 and Gel/Alg-CDH-15-1.5 are much higher than those of Gel/Alg-CDH-10-1.0, indicating strong hydrogen bonding interaction is more favorable for enhancing the shape fidelity. With its excellent printability and shape fidelity, Gel/Alg-CDH-20-2.0 could be printed as orthogonal grids with different filament diameters and different spacing by adjusting the air pressure and model path spacing (Figure 3E).

### The sequential strengthening strategy of Gel/Alg-CDH hydrogels

**Figure 4.**
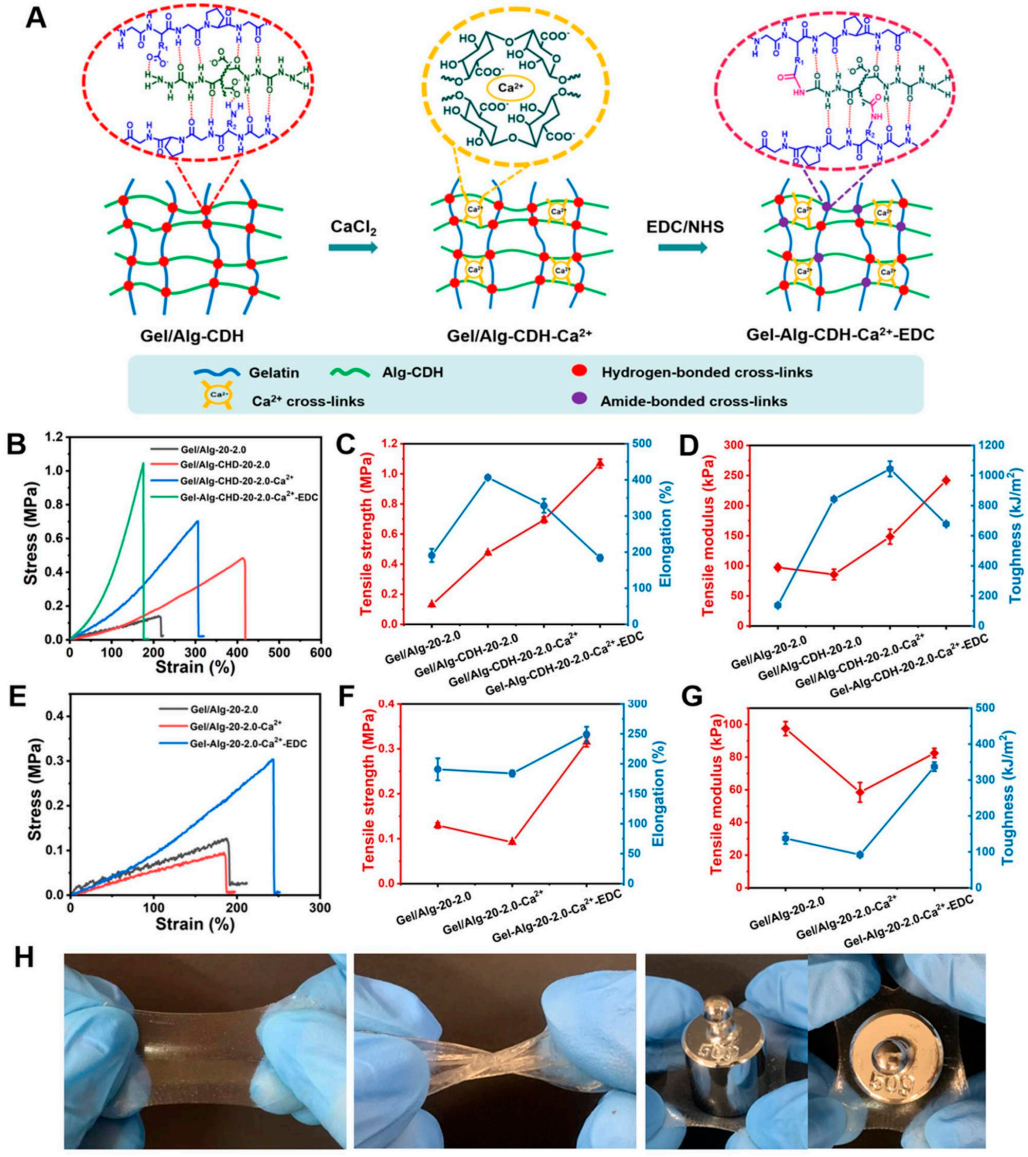
Mechanical properties of Gel/Alg-CDH-20-2.0 hydrogels treated with the sequential strengthening strategy. (**A**) The mechanism of sequential strengthening strategy. The physical crosslinking points of the hydrogen-bonded Gel/Alg-CDH hydrogel is further increased by Ca^2+^ crosslinking, followed by EDC/NHS crosslinking to obtain strong and tough physical–chemical synergistic Gel-Alg-CDH-Ca^2+^-EDC hydrogel. Tensile stress–strain curves of (**B**) Gel/Alg-CDH-20-2.0 hydrogels and (**E**) Gel/alg-20-2.0 hydrogels treated with the sequential strengthening strategy. Tensile strength and elongation at break of (**C**) Gel/Alg-CDH-20-2.0 hydrogels and (**F**) Gel/alg-20-2.0 hydrogels treated with the sequential strengthening strategy. Tensile modulus (10–20% strain) and toughness of (**D**) Gel/Alg-CDH-20-2.0 hydrogels and (**G**) Gel/alg-20-2.0 hydrogels treated with the sequential strengthening strategy. (**H**) Digital photographs of printed Gel-Alg-CDH-20-2.0-Ca^2+^-EDC hydrogel, which could withstand large tensile deformation and twisting deformation, and 300 μm thickness of hydrogel sheet could withstand a load of 50 g.

Although hydrogen bonding strengthened Gel/Alg-CDH demonstrated an excellent printability, it is difficult to obtain high mechanical properties and physiological environmental stability with a hydrogen-bonded crosslinking network alone. Natural soft tissues like corneas tend to exhibit a J-shaped stress–strain behavior under tension, being soft at small strains but hardened exponentially with increasing strain (tensile strength is up to the several MPa). This is mainly attributed to the extracellular matrix collagen fiber network of natural soft tissues and the physical and chemical interactions in the network. However, the homogeneous natural polymer hydrogels, such as gelatin and polysaccharides, typically exhibit linear stress–strain curves with strengths only at the kPa level. Here, we presented the sequentially strengthening strategy via post-printing physical and chemical cross-linking to fabricate Ca^2+^/EDC crosslinked Gel/Alg-CDH (recorded as Gel-Alg-CDH-Ca^2+^-EDC) hydrogel, as shown in Figure 4A. The cross-linked network of Gel/Alg-CDH was mainly formed by the hydrogen bonding interactions between gelatin and Alg-CDH. Firstly, to quantitatively study the interaction strengths among Gel/Alg and Gel/Alg-CDH, Materials Studio was used to simulate the molecular structures and calculate the interaction energies based on DFT, in which gelatin molecule was simplified to the representative sequence Hyp-Pro-Gly, and alginate molecule was simplified to monosaccharide units [43–44]. As shown in Supplementary Figure S7, molecular models show that the hydrazide structures (-CO-NH-NH-CO-NH_2_) of Alg-CDH formed a higher density of hydrogen bonding with amide, -OH (or -NH_2_, -COOH residue) moieties of gelatin. Therefore, the interaction energy of Gel/Alg-CDH (−27.96 kcal/mol) was larger than that of Gel/Alg (−24.33 kcal/mol), indicating that Gel/Alg-CDH forms stronger interactions (Supplementary Table S1). As a result, the hydrogen-bonded crosslinked network of Gel/Alg-CDH was stronger than that of Gel/Alg. As shown in Figure 4B and C, the tensile strength of Gel/Alg-20-2.0 was 0.129 ± 0.006 MPa and the elongation at break was 190.9 ± 18.4%, while the tensile strength of Gel/Alg-CDH-20-2.0 was increased to 0.474 ± 0.011 MPa and the elongation at break reached 406.5 ± 4.5%. The introduction of the hydrazide moieties significantly enhanced the mechanical properties of Gel/Alg-CDH-20-2.0, and its toughness was enhanced 6.1 times compared to Gel/Alg-20-2.0, from 137.6 ± 15.4 to 843.5 ± 8.4 kJ/m^2^ (Figure 4D). It is worth noting that the initial tensile modulus (10%–20% strain) of Gel/Alg-CDH-20-2.0 (85.6 ± 8.7 kPa) was not significantly different from that of Gel/Alg-20-2.0 (97.5 ± 4.3 kPa), and even slightly reduced, as shown in Figure 4D. This indicated that this high-density hydrogen bonding crosslinking was more aimed at improving the toughness of Gel/Alg-CDH-20-2.0, rather than rigidity.

To further improve the mechanical properties of Gel/Alg-CDH-20-2.0 from soft-tough to strong-tough hydrogel, we immersed the printed Gel/Alg-CDH-20-2.0 in low concentration of CaCl_2_ solution (50 mM) and EDC/NHS solution (10 mM) sequentially (Figure 4A). The Ca^2+^ could form ionic crosslinks with the alginate molecular chains in Gel/Alg-CDH-20-2.0 to further increase the physical cross-linking points, while EDC/NHS could catalyze the amide reaction between the aminos and carboxyl groups in gelatin and Alg-CDH to form a chemical cross-linking network. As shown in Figure 4B and C, the tensile strength of Gel/Alg-CDH-20-2.0-Ca^2+^ crosslinked by Ca^2+^ was further increased to 0.693 ± 0.019 MPa, but the elongation at break was reduced to 328.4 ± 19.4%. The tensile modulus of Gel/Alg-CDH-20-2.0-Ca^2+^ was also increased to 148.5 ± 12.4 kPa, and the toughness reached a maximum value of 1044.2 ± 50.9 kJ/m^2^ (Figure 4D). The Gel/Alg-CDH-20-2.0-Ca^2+^ achieved the best toughness, but as a cornea implant, the moderate chemical cross-linking is necessary to provide swelling stability *in vivo*. After chemical crosslinking treatment, the tensile strength of the Gel-Alg-CDH-20-2.0-Ca^2+^-EDC reached a maximum value of 1.068 ± 0.029 MPa, and the elongation at break continued to decrease to 184.1 ± 8.3% but was adequately stretchable. The tensile modulus of Gel-Alg-CDH-20-2.0-Ca^2+^-EDC continued to increase to 242.1 ± 3.6 kPa and the toughness decreased to 677.6 ± 13.7 kJ/m^2^, which was tough enough to withstand suturing (to be shown in the later section). Intriguingly, the Gel-Alg-CDH-20-2.0-Ca^2+^-EDC exhibited a J-shaped stress–strain curve similar to that of natural soft tissues (Figure 4B). Previous studies suggest that natural soft tissues exhibiting J-shaped stress–strain behavior are mainly originated from the hierarchical structure of collagen microfibers. These fibers are oriented under tension and produce nonlinear elasticity [45, 46]. Herein, we speculate that the J-shaped stress–strain behavior of Gel-Alg-CDH-20-2.0-Ca^2+^-EDC hydrogel mainly originates from the hierarchical crosslinked networks, where the rigidity of hydrogen-bonded crosslinked network, ionically crosslinked network and chemically crosslinked network present in the hydrogel is sequentially increased. Therefore, at low strains, the flexible physical cross-linked network mainly bore the load and exhibited a low modulus; at high strains, the rigid chemical cross-linked network started to deform and exhibited increasing modulus with strain, that is, strain–hardening. Although the tensile strength of Gel-Alg-CDH-20-2.0-Ca^2+^-EDC was still lower than that of natural corneas (3–5 MPa) [28], its mechanical properties have far surpassed those of common natural polymer hydrogels (0.1–0.3 MPa) [9, 23, 36]. And with the initial modulus similar to that of natural corneas (150–700 kPa) [47], it is expected to be a biomechanically matched corneal implant.

Controlling the degree of chemical crosslinking is particularly important in the sequential strengthening strategy. Insufficient chemical cross-linking could not maintain the stability of hydrogels, while excessive chemical cross-linking would lead to brittle networks and ultimately deteriorated the toughness of hydrogels. Therefore, we studied the effect of different EDC/NHS crosslinking treatment times (0, 1, 3, 5, 10 min) on the mechanical properties of Gel-Alg-CDH-20-2.0-Ca^2+^-EDC hydrogels. Firstly, the structural parameter crosslink densities (*ν*_c_) of Gel-Alg-CDH-20-2.0-Ca^2+^-EDC hydrogels with different EDC/NHS treatment times were calculated by rheological equation using the rubber-elastic plateau *G*′_p_ [34]. As shown in Supplementary Figure S8, the *G*′_p_ of Gel-Alg-CDH-20-2.0-Ca^2+^-EDC hydrogels gradually increased with the longer chemical crosslinking time, and the crosslink density also increased. Besides, the amide reaction efficiencies (*ξ*) of EDC/NHS catalysis were calculated by determining the amino content of different hydrogels using ninhydrin colorimetry (Supplementary Figure S9). Taking the reaction efficiency of Gel-Alg-CDH-20-2.0-Ca^2+^ hydrogel without chemical cross-linking as 0% (100% amino content), the reaction efficiencies of the Gel-Alg-CDH-20-2.0-Ca^2+^-EDC hydrogels with 1, 3, 5, and 10 min EDC/NHS treatment time were 14.1%, 22.9%, 26.3%, and 33.8%, respectively. These results showed that the hydrogels treated with low concentration (10 mM) EDC/NHS solution exhibited a good controllability to obtain suitable chemical crosslinking density. As shown in Supplementary Figure S10A, with the extension of crosslinking treatment time, the tensile modulus of Gel-Alg-CDH-20-2.0-Ca^2+^-EDC hydrogels gradually increased and the elongation continuously decreased due to the increase of chemical crosslinking density. The peak values of both tensile strength and toughness appeared with the increase of chemical crosslinking density (Supplementary Figure S10B and C). Although chemical crosslinking could further improve the tensile strength, it would inevitably worsen the toughness of hydrogels. Therefore, we choose 3 min as the chemical crosslinking duration for the sequential strengthening strategy to obtain the most suitable mechanical properties and physiological stability. We also investigated the energy dissipation and recovery performance of Gel/Alg-CDH-20-2.0, Gel/Alg-CDH-20-2.0-Ca^2+^ and Gel-Alg-CDH-20-2.0-Ca^2+^-EDC by a loading-unloading cycle (strain: 100%). As shown in Supplementary Figure S11, they all exhibited a significant hysteresis loop in the loading-unloading cycle, and their dissipation energies were 23.0, 32.2  and 62.5 kJ/m^2^, with a recovery rate of 40.4%, 52.5% and 60.8%, respectively. This indicated that the hydrogen bonding cross-linked flexible network of Gel/Alg-CDH hydrogel was easily broken and difficult to recover under a large strain. The Gel/Alg-CDH-Ca^2+^ hydrogel contained increased physical ionic crosslinking points compared to the solely hydrogen bonding crosslinking network, thus generating greater energy dissipations and increased reversibility. Whereas the Gel-Alg-CDH-Ca^2+^-EDC hydrogel consisted of both the sacrificial physical crosslinking network for energy dissipation and the rigid chemical crosslinking network for shape fixation, so it possessed a greater energy dissipation capacity while maintaining a higher reversibility compared to Gel/Alg-CDH and Ge/Alg-CDH-Ca^2+^. As shown in Figure 4H, this physico-chemical crosslinking synergistic Gel-Alg-CDH-20-2.0-Ca^2+^-EDC hydrogel demonstrated excellent mechanical properties, with an ability to withstand a large tensile deformation and torsional deformation, and the 300 μm thickness of the hydrogel sheet could withstand a load of 50 g. In addition, we evaluated the change of the tensile properties of the Gel-Alg-CDH-20-2.0-Ca^2+^-EDC hydrogel in PBS at 37°C. As shown in Supplementary Figure S12, the mechanical properties of the hydrogel significantly decreased on day 3, and the tensile strength decreased to 0.425 ± 0.044 MPa; on day 7, the tensile strength continued to decrease to 0.265 ± 0.056 MPa. This is mainly due to the dissociation of the ionic-crosslinks network of the Gel-Alg-CDH-20-2.0-Ca^2+^-EDC hydrogel.

For comparison, the unmodified Gel/Alg-20-2.0 hydrogel was also subjected to the sequential strengthening strategy. As shown in Figure 4E–G, the mechanical properties of Gel/Alg-20-2.0-Ca^2+^ even deteriorated compared to Gel/Alg-20-2.0, with a relatively lower tensile strength (0.092 ± 0.003 MPa), elongation at break (184.0 ± 5.0%), initial modulus (58.5 ± 6.0 kPa) and toughness (92.1 ± 6.9 kJ/m^2^). This was because Gel/Alg-20-2.0 lacked the original high-density hydrogen bonding cross-links compared to Gel/Alg-CDH, and the large swelling caused by immersion in the low-concentration CaCl_2_ solution deteriorated its mechanical properties, and this weakening effect of swelling exceeded the enhancement effect of Ca^2+^ cross-linking. Although the mechanical properties of Gel-Alg-20-2.0-Ca^2+^-EDC were also improved after chemical crosslinking, e.g. the tensile strength (0.315 ± 0.010 MPa), elongation at break (249.3 ± 12.6%) and toughness (337.1 ± 12.6 kJ/m^2^) were increased compared to Gel/Alg-20-2.0, the mechanical properties of Gel-Alg-20-2.0-Ca^2+^-EDC are far inferior to those of Gel-Alg-CDH-20-2.0-Ca^2+^-EDC. This demonstrated that the formation of high-density hydrogen bonding crosslinks between hydrazide and gelatin in Gel/Alg-CDH is a vital prerequisite for the sequential strengthening strategy to achieve high strength, tough hydrogel and swelling-resistant hydrogel. In addition, as displayed in Supplementary Figure S13A and D, the Gel/Alg-CDH-10-1.0 and Gel/Alg-CDH-15-1.5 also exhibited excellent mechanical properties after the sequential strengthening strategy. The Gel/Alg-CDH-10-1.0-Ca^2+^-EDC exhibited the tensile strength of 0.526 ± 0.025 MPa (Supplementary Figure S13B) and the toughness of 274.8 ± 13.5 kJ/m^2^ (Supplementary Figure S13C); Gel/Alg-CDH-15-1.5-Ca^2+^-EDC exhibited the tensile strength of 0.595 ± 0.021 MPa (Supplementary Figure S13E) and the toughness of 438.6 ± 63.5 kJ/m^2^ (Supplementary Figure S13F). The tensile properties of those hydrogels with the same treatment are stronger with increasing the solid content, as shown in Supplementary Table S2. In addition, the compressive modulus of Gel/Alg-CDH-10-1.0-Ca^2+^-EDC, Gel/Alg-CDH-15-1.5-Ca^2+^-EDC and Gel/Alg-CDH-20-2.0-Ca^2+^-EDC were 103.6 ± 6.0, 241.2 ± 24.2 and 697.7 ± 34.1 kPa, respectively (Supplementary Figure S14).

### Evaluation of transparency, swelling degree, biodegradability and suture retention capacity

**Figure 5.**
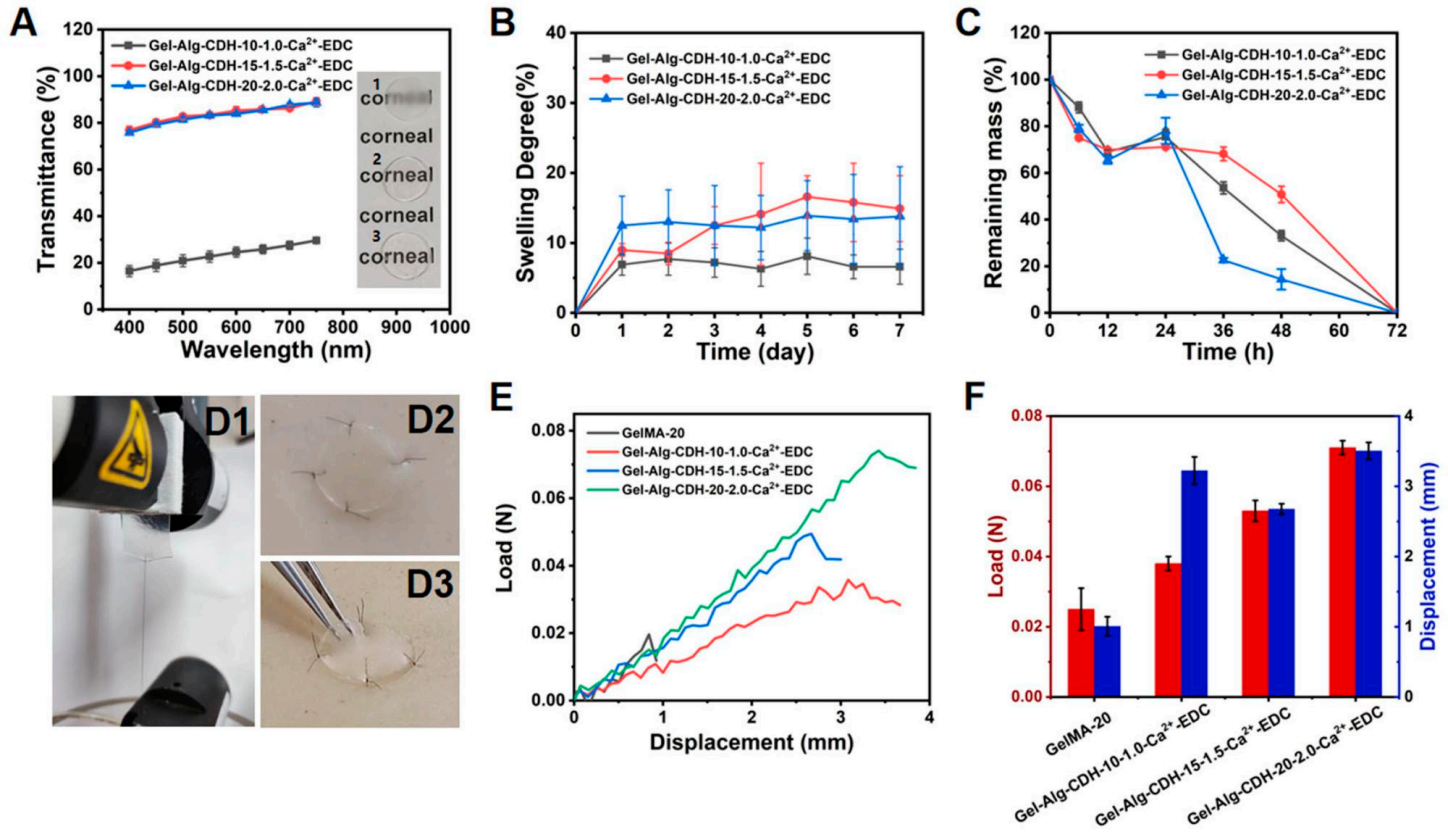
Characterizations of Gel-Alg-CDH-Ca^2+^-EDC hydrogels. (**A**) Light transmittance of the hydrogels on visible light spectrum. The embedded image showed the transparency of hydrogels. (1) Gel-Alg-CDH-10-1.0-Ca^2+^-EDC, (2) Gel-Alg-CDH-15-1.5-Ca^2+^-EDC and (3) Gel-Alg-CDH-20-2.0-Ca^2+^-EDC. (**B**) SD of the hydrogels in PBS at 37°C for 7 days. (**C**) Degradation behavior of the hydrogels in 0.1 U/ml collagenase a solution at 37°C within 72 h. (**D**) Digital images of suture retention test and suturability of hydrogels. (**E**) Load–displacement curves of suture retention test of hydrogels. (**F**) Maximum loads and maximum displacements of suture retention tests of hydrogels.

We then evaluated several key indexes of Gel-Alg-CDH-Ca^2+^-EDC hydrogels as cornea implants, including transparency, SD, enzymatic degradability and suturability properties. The transmittance of Gel-Alg-CDH-Ca^2+^-EDC hydrogels was measured in the wavelength range of 400–750 nm using a UV-vis spectrophotometer. As shown in Figure 5A, Gel-Alg-CDH-20-2.0-Ca^2+^-EDC and Gel-Alg-CDH-15-1.5-Ca^2+^-EDC exhibited good transparency, and their light transmittance at 500 mm wavelength was 81.5 ± 1.2% and 82.8 ± 1.3%, respectively, which could meet the transparency requirements of cornea scaffolds (transmittance of natural cornea: 89%) [28]. However, Gel-Alg-CDH-10-1.0-Ca^2+^-EDC exhibited low transparency, with a light transmittance of only 20.9 ± 2.6% at 500 mm wavelength, suggesting the formation of aggregates of gelatin and Alg-CDH at low concentrations.

The SD is also an important property of cornea implants. In cornea transplantation, a low SD maintains a good anastomosis between the graft and the recipient cornea, reducing postoperative cornea deformation and pressure on the eye from the implant. Hydrogels were immersed in phosphate buffer (PBS) and incubated at 37°C to simulate their swelling behavior *in vivo*. All the three hydrogels reached a swelling equilibrium after 1 day, and the slight fluctuation of SDs was observed over the next 7 days (Figure 5B). The SDs of Gel-Alg-CDH-20-2.0-Ca^2+^-EDC and Gel-Alg-CDH-15-1.5-Ca^2+^-EDC at day 7 were 14.9 ± 4.7% and 13.8 ± 7.1%, respectively, which were very close to that of the natural cornea (14.5 ± 0.6%) [28]. The opaque Gel-Alg-CDH-10-1.0-Ca^2+^-EDC had a lower equilibrium swelling rate (6.6 ± 2.5%), indicating that the aggregates formed at low concentrations reduced the water permeability. Enzymatic degradability is another important performance indicator of corneal regeneration scaffolds. We exposed the hydrogels to collagenase A solution at 37°C to investigate their *in vitro* enzymatic degradation behavior. As depicted in Figure 5C, there was a rapid *in vitro* degradation behavior of all the three hydrogels within 72 h. The remaining mass percentages of Gel-Alg-CDH-10-1.0-Ca^2+^-EDC, Gel-Alg-CDH-15-1.5-Ca^2+^-EDC and Gel-Alg-CDH-20-2.0-Ca^2+^-EDC at 48 h were measured to be 33.1 ± 2.2%, 50.8 ± 3.5% and 14.4 ± 4.4%, respectively. And the rapid enzymatic degradation is necessary in superficial corneal injury to achieve rapid tissue integration and regeneration [48].

The suturability of hydrogel scaffolds is critical for the surgical operation of the keratoplasty. GelMA hydrogels are readily available and have excellent biocompatibility, making them the most widely studied cornea scaffolds. However, they are weak and brittle and do not have sufficient mechanical properties to resist surgical sutures. Here, we assessed the suturability of Gel-Alg-CDH-Ca^2+^-EDC hydrogels by measuring their suture retention capacity, with the 20% GelMA hydrogel as a control group. Briefly, a 10-0 ophthalmic suture was passed through the hydrogel and the maximum load and displacement of the suture during stretching was tested (Figure 5D1). The 20% GelMA hydrogel had almost no suture retention capacity, and the suture cut through the hydrogel at a very small load (0.025 N), and the deformation distance was only about 1 mm (Figure 5E and F). In contrast, the physico-chemical synergistically crosslinked Gel-Alg-CDH-Ca^2+^-EDC hydrogel demonstrated a much better suture retention capacity and could withstand a higher load and larger displacement. Gel-Alg-CDH-10-1.0-Ca^2+^-EDC could withstand a load of 0.038 N and a displacement of 3.2 mm, Gel-Alg-CDH-15-1.5-Ca^2+^-EDC could withstand a load of 0.053 N and a displacement of 2.7 mm, and Gel-Alg-CDH-20-2.0-Ca^2+^-EDC could withstand a load of 0.071 N and a displacement of 3.5 mm. The maximum load and maximum displacement of Gel-Alg-CDH-20-2.0-Ca^2+^-EDC were ∼3-fold as those of the 20% GelMA hydrogel, hinting that it could withstand sharp piercing during surgical suture without breaking. Figure 5D2 and D^3^ shows that we could successfully suture the hydrogel onto the skin model *in vitro*.

Considering all the above indicators, we concluded that Gel-Alg-CDH-20-2.0-Ca^2+^-EDC was the most suitable as a cornea implant, and it was chosen for the subsequent experiments.

### Construction of 3D-printed rhEGF/TSA-loaded bilayer hydrogel scaffold and biological properties assessment

**Figure 6.**
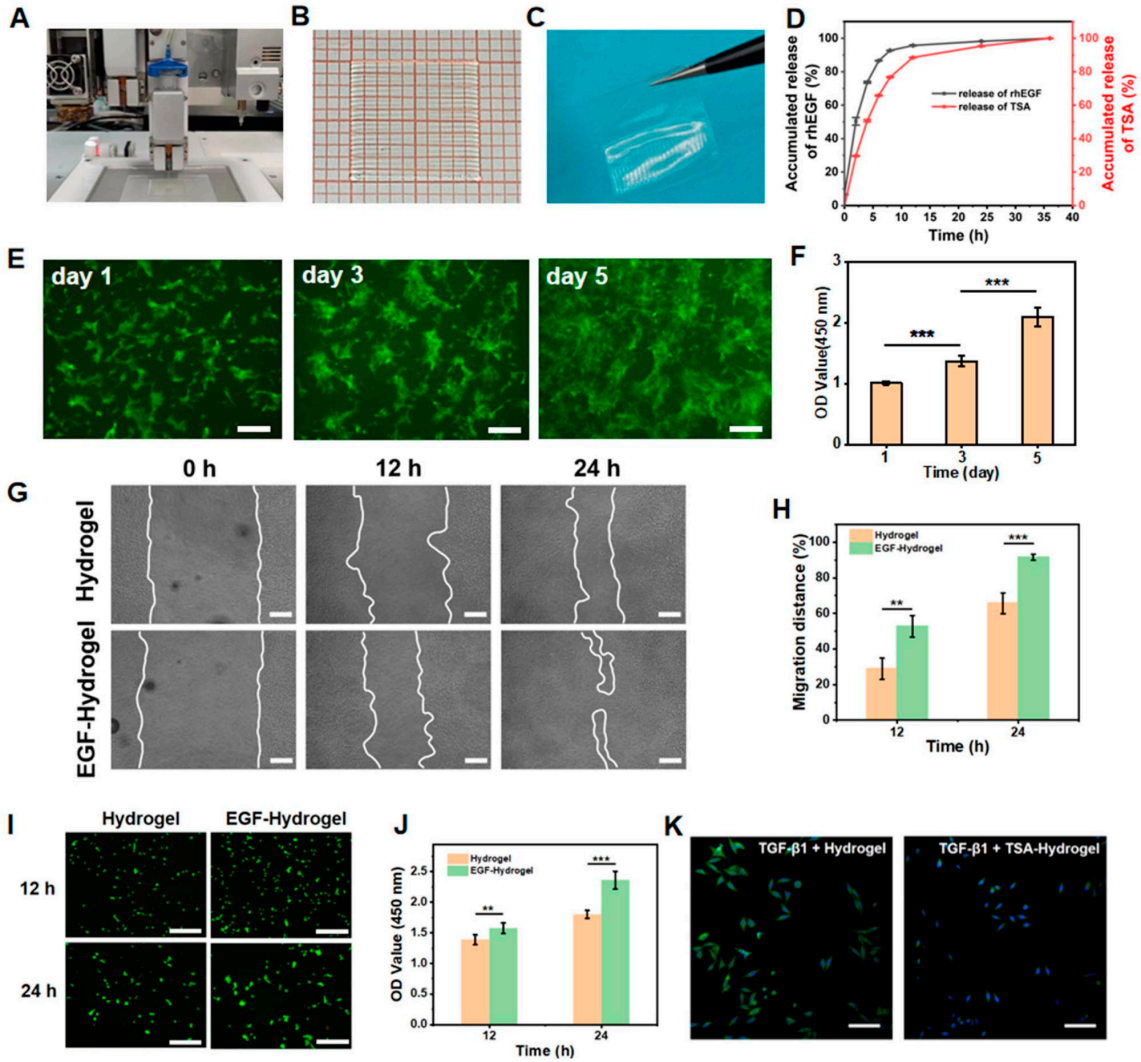
Preparation of the rhEGF/TSA bilayer hydrogel scaffold and bioactivity evaluation of EGF-loaded hydrogels and TSA-loaded hydrogels. Data are expressed as mean ± standard deviation (*n* ≥ 4; **P < 0.01, ***P < 0.001). (**A**) Preparation of the rhEGF/TSA bilayer hydrogel scaffold using a multi-nozzle printing system. (**B**) Digital image of printed rhEGF/TSA bilayer hydrogel scaffold (scale bars: 1 mm). (**C**) Digital image of printed rhEGF/TSA bilayer hydrogel scaffold treated with the sequential strengthening strategy. (**D**) Cumulative release percentages of rhEGF and TSA from printed Gel-Alg-CDH-20-2.0-Ca^2+^-EDC hydrogel. (**E**) Live/dead staining of rASCs cultured on Gel-Alg-CDH-Ca^2+^-EDC hydrogel for 1, 3, and 5 days, respectively. Scale bars: 500 μm. (**F**) CCK-8 assays of cultured rASCs after 1, 3, and 5 days. (**G**) Migration of rCECs treated with hydrogels and rhEGF-loaded hydrogels in the scratched area at 12 and 24 h. Scale bars: 150 μm. (**H**) Quantification of relative migration distance in the scratched area of the hydrogel group and rhEGF-loaded hydrogel group. (**I**) Live/dead staining images of rCECs treated with hydrogels and rhEGF-loaded hydrogels at 12 and 24 h. Scale bars: 300 μm. (**J**) CCK-8 assays of the cultured rCECs at 12 and 24 h. (**K**) Immunofluorescence staining of α-SMA/DAPI in the TGF-β1-mediated rCECs fibrosis treated with hydrogels and TSA-loaded hydrogels. Scale bars: 100 μm.

The regeneration of cornea defects usually involves the repair of the epithelial and stromal layers, such as ALK. In this work, 3D-printed biomimetic rhEGF/TSA-loaded bilayer hydrogel scaffold was fabricated using Gel/Alg-CDH-20-2.0 as ink and subjected to sequential strengthening strategy. Specifically, rhEGF/TSA-loaded bilayer hydrogel scaffold was obtained by layer-by-layer printing of rhEGF-loaded ink A and TSA-loaded ink B in a multi-nozzle printing system, as shown in Figure 6A and Supplementary Movie S2. The printed bottom layer hydrogel and top layer hydrogel were well fused due to cooling-induced hydrogen bonding network reconstruction at the interface, and the extruded filaments were closely aligned with each other, forming a flat and smooth bilayer hydrogel scaffold (Figure 6B and C). The thickness of the bilayer hydrogel scaffold could be adjusted by selecting specific printing parameters (air pressure, nozzle movement speed and path spacing, etc.). As shown in Supplementary Table S3, controlling other parameters constant, the thickness of bilayer hydrogel scaffolds could be adjusted between 250 and 400 μm by changing the printing air pressure of the bottom and top layers. The adjustable thickness could adapt to different corneal damage depths. Furthermore, rhEGF can stimulate the proliferation and motility of corneal epithelial cells *in vitro*, and has been shown to enhance wound healing in primate models and clinical trials [49, 50]. TSA, a histone deacetylase inhibitor, has been shown to suppress TGF-β1-induced fibrogenesis and decreased corneal haze *in vivo* [51]. So, the rhEGF-loaded top layer hydrogel was expected to promote rapid re-epithelialization of the cornea epithelial layer, while the TSA-loaded bottom layer hydrogel was used to inhibit scar formation during cornea stromal repair. We then tested the release behaviors of rhEGF and TSA from the hydrogels for 36 h in PBS at 37°C. As shown in Figure 6D, both rhEGF and TSA-loaded hydrogels immersed in excess solution exhibited an initial rapid release, as described in the literature [52], with their release reaching 92.7 ± 0.06% and 76.7 ± 0.26% at 8 h, respectively, and then the release levelled off. This confirmed that loaded rhEGF and TSA could be effectively released from Gel-Alg-CDH-20-2.0-Ca^2+^-EDC hydrogels and that bilayer hydrogel scaffolds could be used for delivery of active factors at sites of corneal injury.

To explore the application of rhEFG/TSA bilayer hydrogel scaffolds in corneal regeneration, we first evaluated the biocompatibility of Gel-Alg-CDH-20-2.0-Ca^2+^-EDC hydrogels. The rASCs were cultured on hydrogels and then assayed for viability and proliferative capacity by live/dead staining and CCK-8 assay on days 1, 3 and 5 (Figure 6E and F). Live/dead staining demonstrated that rASC grew well on the hydrogel surface, maintained high activity, and the cells showed significant proliferation at day 3 and day 5, with a gradual increase in the OD value of the CCK8 assay. The results demonstrated that the Gel-Alg-CDH-20-2.0-Ca^2+^-EDC hydrogel could support cell adhesion and proliferation. Then, we evaluated the bioactivity of rhEGF-loaded hydrogel and TSA-loaded hydrogel, respectively. We verified the promotion of cell migration and proliferation by rhEGF release from rhEGF-loaded hydrogels, while pristine hydrogels were employed as the control groups. As shown in Figure 6G and H, scratch healing was significantly increased in the rhEGF-hydrogel group compared to the hydrogel group at different time points (*P* < 0.01). The quantitative migration distance of scratch closure was significantly accelerated in rCECs treated with rhEGF-hydrogel at 12 and 24 h. The migration rates of the pristine hydrogel and rhEGF-hydrogel groups were 65.7% and 91.6% after 24 h, respectively, indicating that the release of rhEGF significantly enhanced the migration ability of rCEC cells. In addition, the release of rhEGF also promoted the proliferation of rCEC. Figure 6I and J exhibits that rCEC in the rhEGF-hydrogel group had better proliferative capacity in serum-free culture. The OD value of CCK8 assays in the rhEGF-hydrogel group was 1.13-fold and 1.31-fold as that in the pristine hydrogel group at 12 and 24 h, respectively. And rhEGF-hydrogel group also showed a higher cell density in live/dead staining at these two time points.

We also validated the biocompatibility of TSA-load hydrogel and its inhibition on fibrosis. Firstly, the cytotoxicity test of TSA-load hydrogel was performed using live/dead staining and CCK8 assay. As shown in Supplementary Figure S15, both the hydrogel-treated and TSA-hydrogel-treated rASCs maintained a good activity and proliferation capacity during the 3 days incubation. Although the OD values of CCK8 assays were slightly lower in the TSA-hydrogel group than that of pristine hydrogel group, no significant differences were observed. These results demonstrated that TSA-loaded hydrogels still maintained good biocompatibility. Furthermore, the effect of hydrogel-released TSA on TGF-β1 mediated myofibroblast phenotype was assessed by α-SMA/DAPI immunofluorescence staining with mouse fibroblasts (L929). As shown in Figure 6K, the expression of α-SMA protein was significantly reduced in the TSA-loaded hydrogel-treated group, suggesting that TSA-loaded hydrogel could inhibit the conversion of fibroblast phenotype to myofibroblast phenotype and reduce the excessive synthesis of collagen fibers, which is expected to reduce corneal scarring in corneal repair.

*In vitro* results indicated that the 3D-printed rhEFG/TSA-loaded biomimetic bilayer scaffold could promote re-epithelialization and inhibit corneal scar formation, which is expected to target the different needs of the epithelial and stromal layers during corneal regeneration to achieve an ideal repair effect.

### *In vivo* assessment of 3D-printed rhEGF/TSA-loaded bilayer hydrogel scaffold in the rabbit ALK model

**Figure 7.**
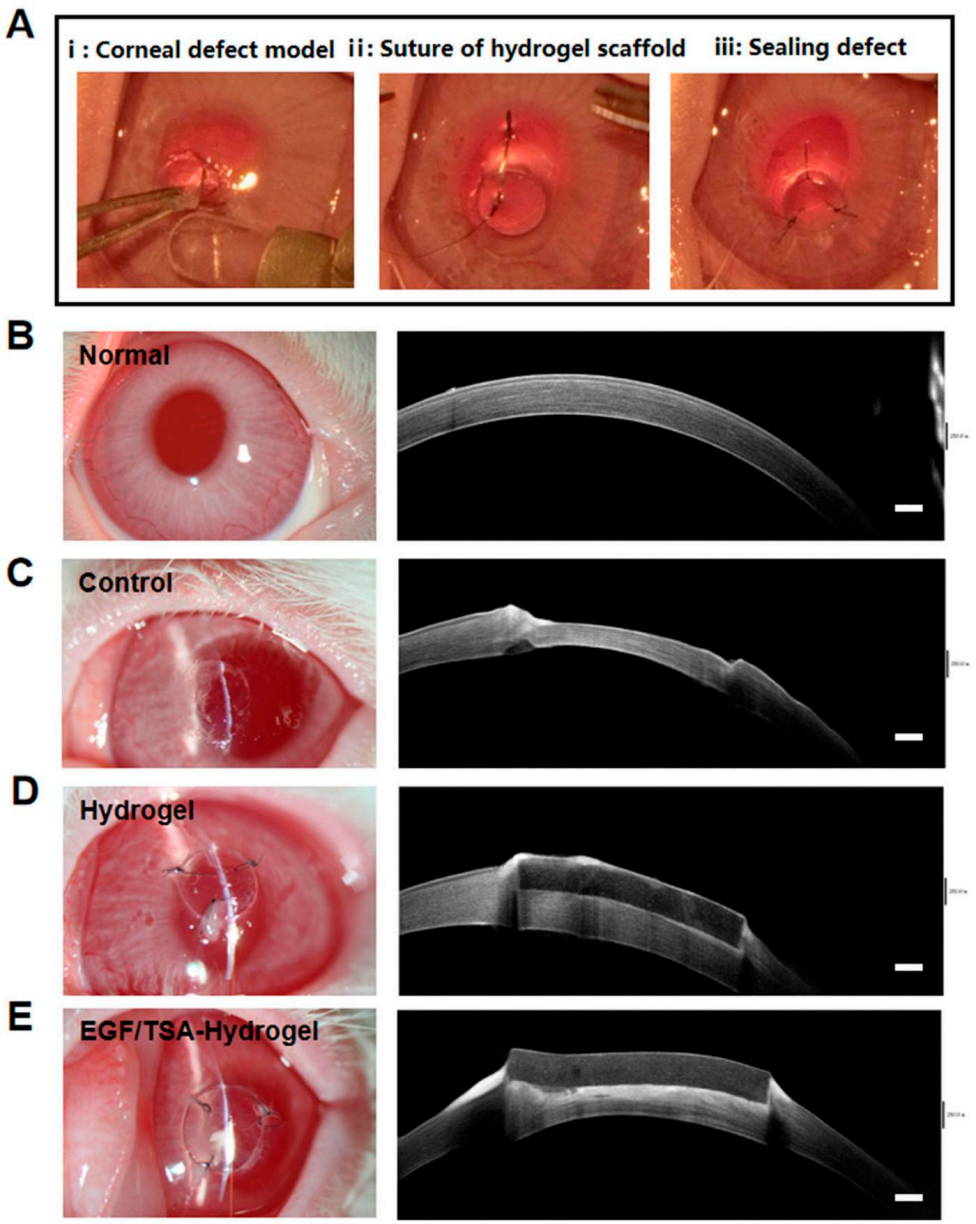
*In vivo* application of 3D-printed rhEGF/TSA bilayer hydrogel scaffold in rabbit ALK model. (**A**) Surgical procedures of ALK, including removal of the corneal epithelium and part of the corneal stroma, implantation of the hydrogel scaffold and surgical suture. (**B–E**) Representative slit lamp (left) and AS-OCT (right) images of the normal cornea, the cornea defect group (control group), the hydrogel implantation group, the rhEGF/TSA bilayer hydrogel implantation group, respectively. Scale bars of AS-OCT images: 250 μm.

Herein, the rhEGF/TSA-loaded bilayer hydrogel scaffold was applied to the New Zealand rabbit corneal ALK model and its biocompatibility and biointegration for sealing and repairing corneal defects was evaluated. The surgical procedure is shown in Figure 7A. The surgeon injured the cornea with a customized 3 mm trephine, removed a certain depth of anterior lamella, and then placed the 3D-printed hydrogel scaffold or rhEGF/TSA-loaded bilayer hydrogel scaffold over the damaged area. Finally, these hydrogel scaffolds were sutured directly to the surrounding cornea tissue with a 10-0 suture, with the good suturing-resistant ability. The control group only produced corneal defects without hydrogel implantation or surgical suture. Figure 7B–D shows that the defect depth generated in this study was about 1/3 of the corneal thickness through slit lamp and AS-OCT observation. The sutured hydrogel scaffold and rhEGF/TSA-loaded bilayer hydrogel scaffolds all maintained a good optical transparency under the slit-lamp microscope, and the AS-OCT image further showed that they attached well on the cornea receptor bed and sealed the cornea defect (Figure 7C and D). And the sutured scaffold could withstand continuous blinking and water flow impact (Supplementary Movie S3). It should be mentioned that due to the limitation of the hydrogel printing needle (23 G, 0.34 mm), the thickness of 3D-printed scaffold was higher than the depth of corneal injury, and the implanted bilayer scaffold was ultimately higher than the normal epithelium. This phenomenon may have adverse effects on corneal epithelialization, therefore requiring the scaffold to have a rapid biodegradation. While our corneal scaffold with rapid degradation rate just met this requirement.

**Figure 8.**
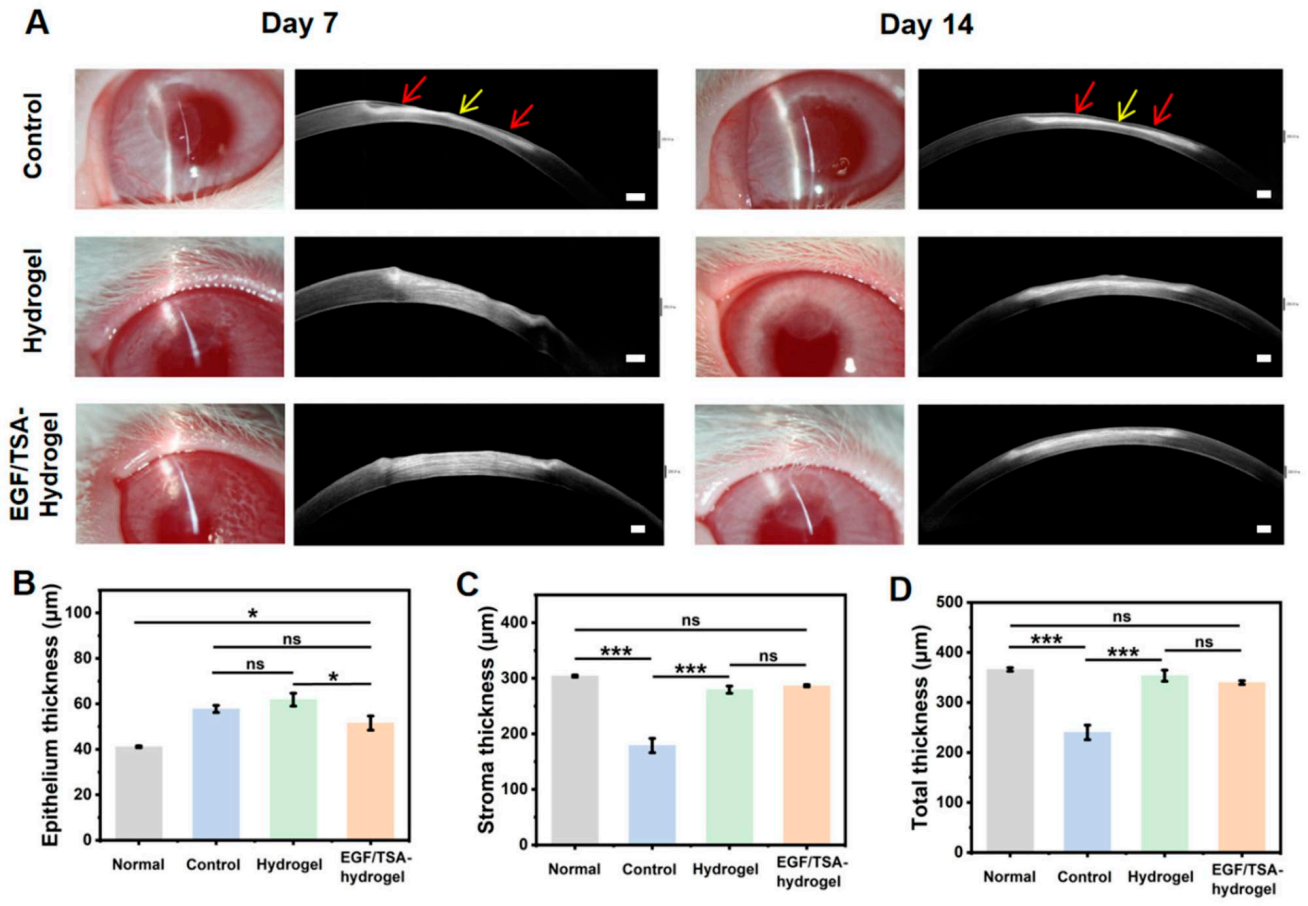
Postoperative observation of corneas post-operation. Data are expressed as mean ± standard deviation (*n* = 3; *P < 0.05, ***P < 0.001, ns: no significance). (**A**) Representative slit lamp and AS-OCT images of the untreated control corneas and the experimental corneas implanted with the hydrogel and rhEGF/TSA bilayer hydrogel on the day 7 and day 14 post-operation. (**B**) Epithelium thickness, (**C**) stroma thickness and (**D**) total thickness of the normal cornea, the untreated control corneas and the experimental corneas implanted with the hydrogel and rhEGF/TSA bilayer hydrogel on day 14 post-operation. Scale bars of AS-OCT images: 250 μm.

We followed up the cornea repair effect of each group on day 7 and day 14 post-operation, as shown in Figure 8A. On the day 7 post-operation, a new epithelium (red arrow) appeared in the cornea defect area in the control group, but the cornea stromal thickness in the central defect area (yellow arrow) was significantly thinner than that of surrounding tissue. In the hydrogel scaffold group and rhEGF/TSA-loaded hydrogel scaffold group, these two kinds of hydrogel scaffolds were integrated with the surrounding tissue and there was no clear boundary between hydrogel scaffolds and the cornea receptor bed. This implied that the implanted hydrogel scaffolds were rapidly biodegraded. Although no new epithelial layer was observed in the cornea defect area in the hydrogel group and the rhEGF/TSA-loaded hydrogel group, the degraded hydrogel filled the cornea stroma, and the thickness of the corneal stroma in the central defect was thicker than that of the control group. On day 14 post-operation, the cornea defect area in the control group had achieved re-epithelialization, forming a complete epithelial layer (red arrow). However, the cornea stromal was not completely repaired, and the thickness of the stroma in the central area (yellow arrow) was still thinner than the surrounding cornea tissue. We quantitatively analyzed the cornea thickness of the central defect area on day 14 post-operation through AS-OCT images (Figure 8B–D). In the control group, the thickness of the epithelial layer (57.80 ± 1.57 μm) was slightly higher than that of the normal cornea (41.1 ± 0.4 μm), but the thickness of the stromal layer (179.0 ± 12.9 μm) was significantly lower than that of the normal cornea (303.97 ± 1.83 μm), so the total cornea thickness (240.2 ± 14.6 μm) was lower than that of the normal cornea (366.1 ± 3.6 μm). Cornea defects in the hydrogel group and rhEGF/TSA-loaded hydrogel group had also achieved re-epithelialization on day 14, and the thickness of the cornea stroma in the hydrogel group (279.6 ± 6.6 μm) and the rhEGF/TSA-loaded hydrogel group (286.6 ± 1.9 μm) were equivalent to that of normal cornea due to the implantation of scaffolds. It is worth noting that the regenerated epithelial layer of the hydrogel group (61.9 ± 2.8 μm) and the control group is thicker than that of the normal cornea. And the repaired cornea defect in the rhEGF/TSA-loaded hydrogel group had the closest morphology to the normal cornea, with the thickness of the regenerated epithelium (51.6 ± 3.1 μm) only slightly higher than that of the normal cornea and the thickness of the stroma comparable to that of the normal cornea.

**Figure 9.**
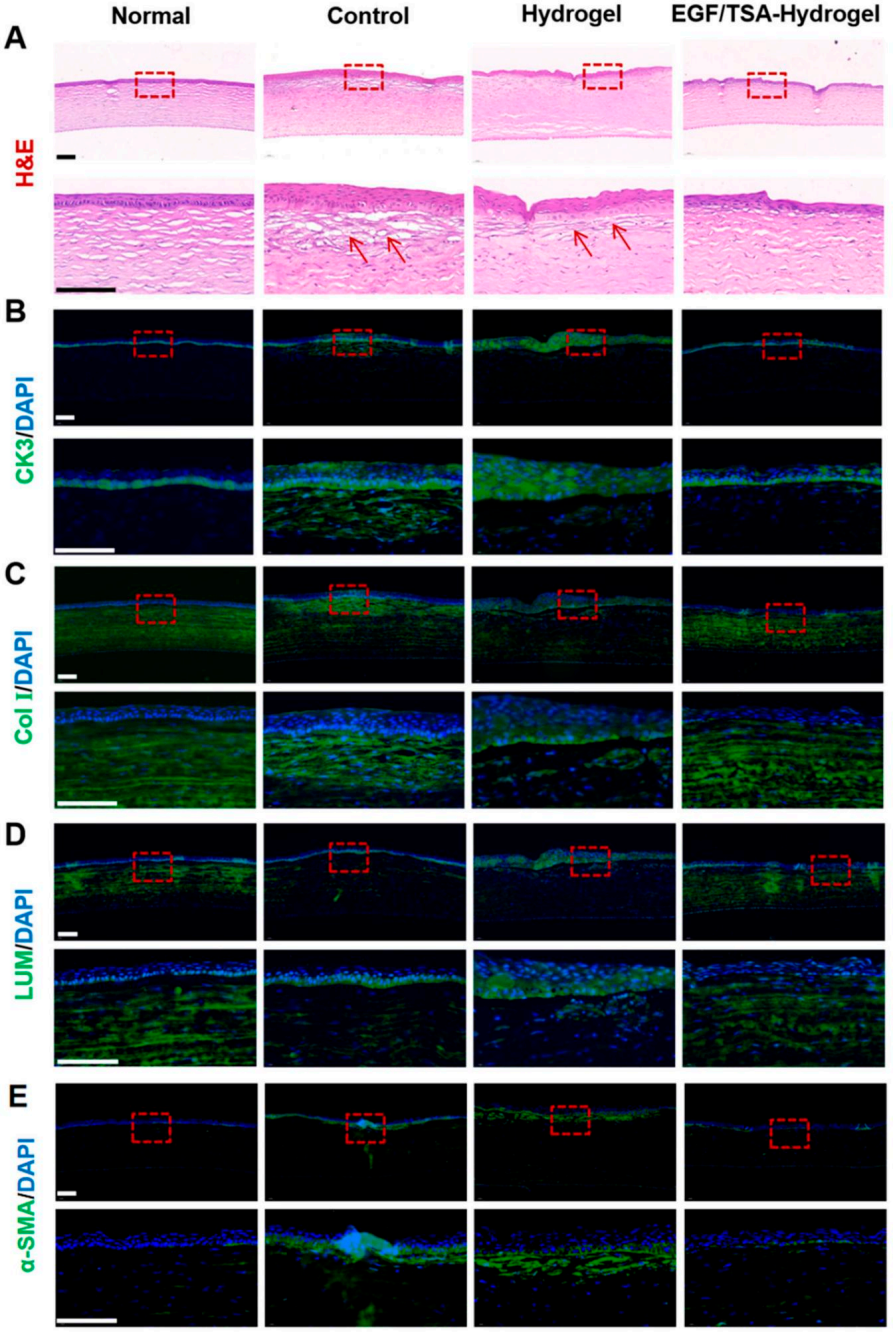
Histological and immunofluorescence analysis of corneas on the day 14 post-operation. (**A**) H&E staining and immunofluorescence staining of (**B**) CK3/DAPI, (**C**) Col I/DAPI, (**D**) LUM/DAPI and (**E**) α-SMA/DAPI of corneas on day 14 post-surgery. The images below are the enlarged views of the rectangular area. Scale bar: 100 μm.

H&E staining and immunofluorescence staining were conducted on day 14 post-operation to further investigate the repair effect of corneal defect in each group, as shown in Figure 9A–E. The control group, hydrogel group and rhEGF/TSA-loaded hydrogel group all formed an integrated epithelium-stroma structure resembling normal corneal tissue (Figure 9A). H&E staining showed that the thickness of the epithelial layer was higher in the control group and hydrogel group than that of the normal group, while the thickness of the epithelial layer in the rhEGF/TSA hydrogel group was comparable to that of the normal tissue, which was consistent with the results of AS-OCT. And epithelial cells in the epithelial layer of the rhEGF/TSA-loaded hydrogel group were more tightly aligned and morphologically closer to that of the normal group, which might result from the regulatory effect of the rhEGF-loaded in the top hydrogel layer. Similarly, all groups regenerated a new corneal stroma consisting of collagen fibers. However, in the control group, the collagen fiber network of the regenerated stroma (red arrow) was disorganized rather than aligned in parallel as that of the normal corneal stroma. This is because that keratinocytes were stimulated by corneal injury to transform into a fibroblast/myofibroblast phenotype, which led to excessive deposition of collagen, forming a disorganized collagen fiber network and even irreversible corneal scars [53]. Although no obvious disordered collagen fibers network was formed, scar tissue with slender dark nuclei (red arrow) appeared in the hydrogel group. The rhEGF/TSA-loaded hydrogel group regenerated a parallel-aligned collagen fiber network similar to that of normal corneal tissue, and only very few random collagen fibers were observed in the area closest to the epithelium. This demonstrated that hydrogel implantation inhibited scar formation during corneal repair, especially with the rhEGF/TSA-loaded bilayer hydrogel scaffold. CK3 (corneal epithelial specific protein) immunofluorescence staining confirmed again that all groups of corneas achieved re-epithelialization and their epithelial layers all expressed CK3 protein (Figure 9B). Immunofluorescence staining of Col I further demonstrated the secretion of a large number of random collagen fibers in the control group, while the bilayer hydrogel group produced parallel collagen fibers network similar to that of the normal cornea (Figure 9C). In addition, LUM (abundantly expressed by corneal keratocyte) and alpha-smooth muscle actin (α-SMA, expressed by myofibroblasts, resulting in abnormal expression of collagen and scar tissue formation) immunofluorescence stainings were conducted to investigate the keratocyte phenotype in defect area (Figure 9D and E). Results showed that LUM was abundantly expressed in the corneal stroma of the bilayer hydrogel scaffold group, which was similar to that of normal cornea, while lower fluorescence intensity was observed in the control group and hydrogel scaffold group. In contrast, α-SMA expression was extremely low in the corneal stroma of the bilayer hydrogel scaffold group, whereas significant α-SMA expression was observed in the control and hydrogel scaffold groups. This demonstrated that rhEGF/TSA-loaded bilayer hydrogel scaffold could inhibit the conversion of keratinocytes to fibroblasts/myofibroblasts and prevent the formation of corneal scarring.

Animal experiment results indicated that Gel-Alg-CDH-20-2.0-Ca^2+^-EDC hydrogels possessed sufficient mechanical properties for resisting surgical sutures, as well as good biocompatibility and biodegradability to support corneal epithelial and stromal regeneration. In particular, a 3D-printed rhEGF/TSA-loaded bilayer hydrogel scaffold could modulate epithelial repair and inhibit scar tissue formation through loaded active factors. It should be pointed out that corneal repair and scar formation are quite complex processes involving the regulation of multiple cytokines and growth factors [54]. Here, we provide a promising scenario of 3D-printed biomimetic bilayer hydrogel scaffolds for corneal regeneration, and the deeper mechanism by which 3D-printed rhEGF/TSA bilayer hydrogel scaffolds exert their bioactive effects remains to be further elucidated. In addition, the currently investigated Gel-Alg-CDH-20-2.0-Ca^2+^-EDC hydrogel scaffolds have a rapid degradation rate and are suitable for ALK models with superficial wounds. For other deep corneal defect models, such as deep ALK, it is necessary to extend the EDC/NHS crosslinking time during the strengthening treatment of hydrogel scaffolds to obtain an appropriate degradation rate for long-term implantation.

## Conclusions

In summary, we developed a hydrogen-bonding strengthened Gel/Alg-CDH ink through simple concentrated solution blending, affording a high and direct printability with appropriate rheological properties such as gel–sol transition temperature, thickened viscosity and shear thinning. The sequential strengthening strategy, that is, first Ca^2+^ crosslinking and then moderately chemical cross-linking contributed to the formation of high-strength and tough Gel-Alg-CDH-Ca^2+^-EDC hydrogels that matched the J-shaped stress–strain behavior of natural soft tissues (low modulus at low strain, high modulus at high strain). The Gel-Alg-CDH-Ca^2+^-EDC hydrogels exhibited high transparency, physiological swelling stability and rapid enzymatic degradability, as well as suturability. The rhEGF/TSA-loaded bilayer hydrogel scaffold was customized using rhEGF-loaded ink A and TSA-loaded ink B via a multi-nozzle printing system, targeting the different needs of epithelial and stromal layer regeneration. In the rabbit ALK model, the rhEGF/TSA-loaded hydrogel scaffold resulted in the formation of integrated epithelium-stroma structure resembling normal corneal tissue by rhEGF-facilitated regulatory and TSA-inhibited scar formation during cornea repair. Development of Gel/Alg-CDH inks and the sequential strengthening strategy provides an ideal option for printing high-strength and tough corneal scaffolds, and this idea can be extended to construct a variety of high strength wholly natural polymer hydrogels due to its simplicity and versatility, offering a new avenue to develop high strength natural polymer hydrogel inks for customizing personalized soft tissue engineering scaffolds.

## Supplementary Material

rbae012_Supplementary_Data

## Data Availability

All of the data reported in this work are available upon request.
