# Supplementary material for: 3D printing sequentially strengthening high-strength natural polymer hydrogel bilayer scaffold for cornea regeneration
Source: Regen Biomater. 2024 Feb 9;11:rbae012. doi: 10.1093/rb/rbae012 (PMC10918636; doi:10.1093/rb/rbae012)
Supplement: rbae012_Supplementary_Data [file rbae012_supplementary_data.zip › Nie Xiongfeng-SI-2023.01.29.docx]

**Supporting Information**

**3D printing sequentially strengthening high-strength natural polymer hydrogel bilayer scaffold for cornea regeneration**

Xiongfeng Nie ^a^, Yong Tang ^b^, Tengling Wu ^a^, Xinrui Zhao ^a^, Ziyang Xu ^a^, Rong Yang ^a^, Yage Sun ^a^, Bin Wu ^b^, Quanhong Han ^b^, Jingwen Hui ^b,^*, Wenguang Liu ^a,^*

*^a^ School of Material Science and Engineering, Tianjin Key Laboratory of Composite and Functional Materials, Tianjin University, Tianjin 300350, China*

*^b^ Tianjin Eye Hospital, Tianjin Key Lab of Ophthalmology and Visual Science, Tianjin Eye Institute,* *Gansu Road 4, Heping District, Tianjin 300020, China*

**Corresponding author (E-mail: [drhuijw@163.com](mailto:hanquanhong126@126.com); [wgliu@tju.edu.cn](mailto:wgliu@tju.edu.cn))*


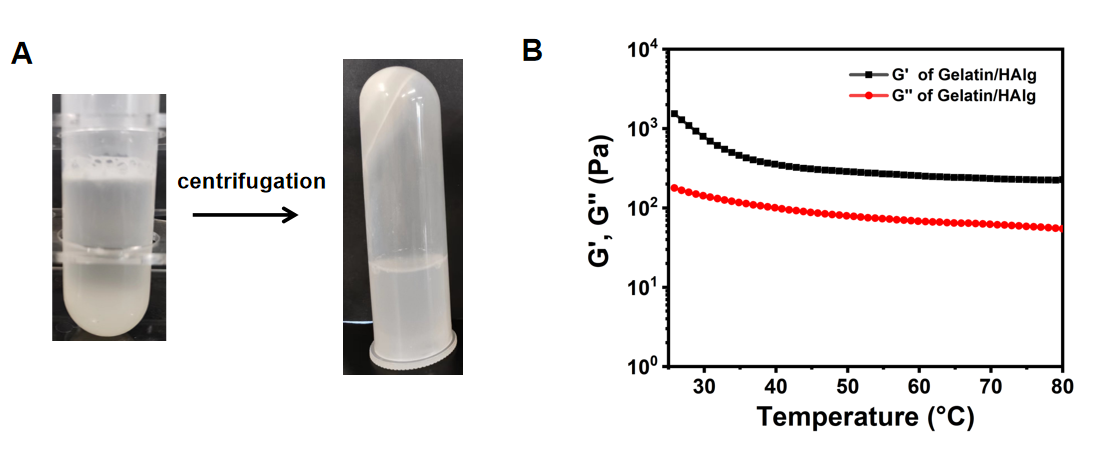


**Fig. S1.** (A) Preparation of Gelatin/HAlg hydrogel. (B) Storage modulus (G') and loss modulus (G'') curves of Gelatin/HAlg hydrogel as a function of temperature.


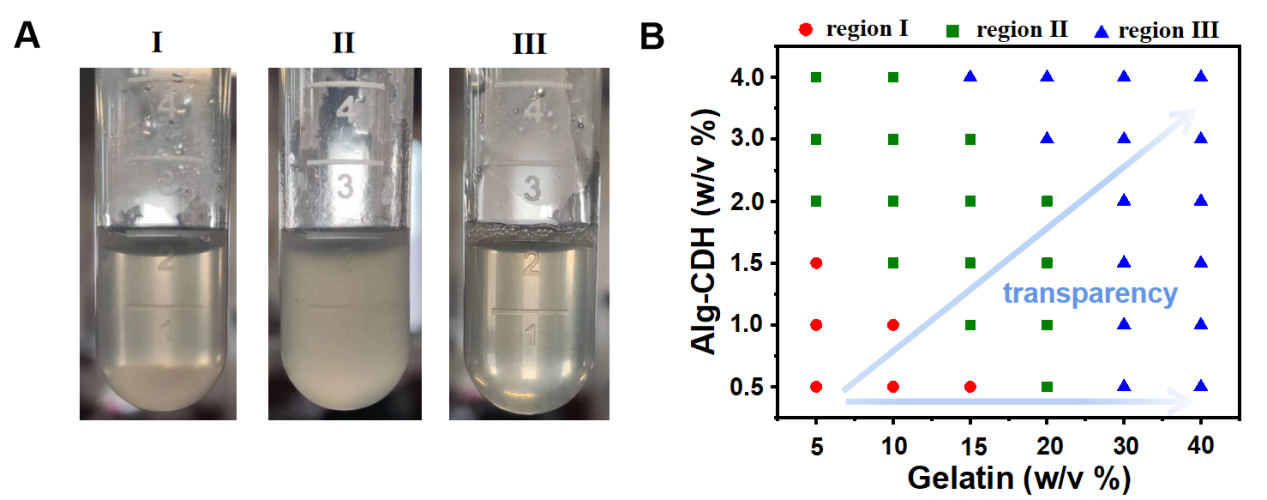


**Fig. S2.** (A) Three different states of the blends: Ⅰ precipitation, Ⅱ opaque homogeneous mixture, Ⅲ transparent homogeneous mixture. (B) Binary diagram of the blends of high concentration gelatin solution and high concentration Alg-CDH solution.


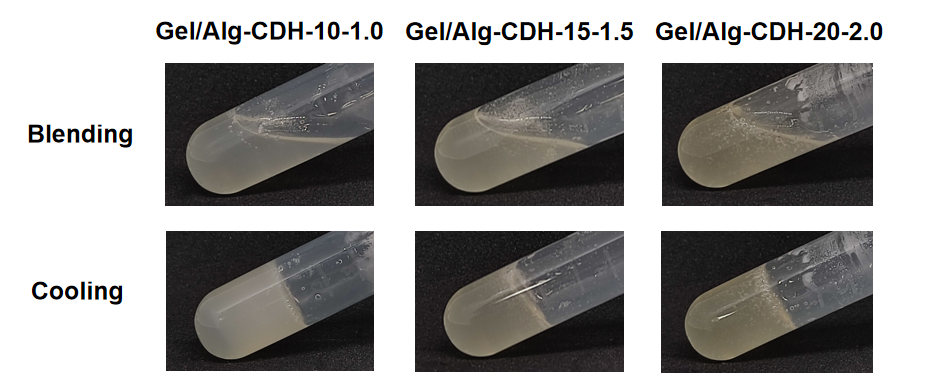


**Fig. S3.** Digital images of the sol-gel transition process of Gel/Alg-CDH-10-1.0, Gel/Alg-CDH-15-1.5 and Gel/Alg-CDH-20-2.0. Gelatin and Alg-CDH were blended to form the homogeneous inks, then hydrogels were formed after cooling.


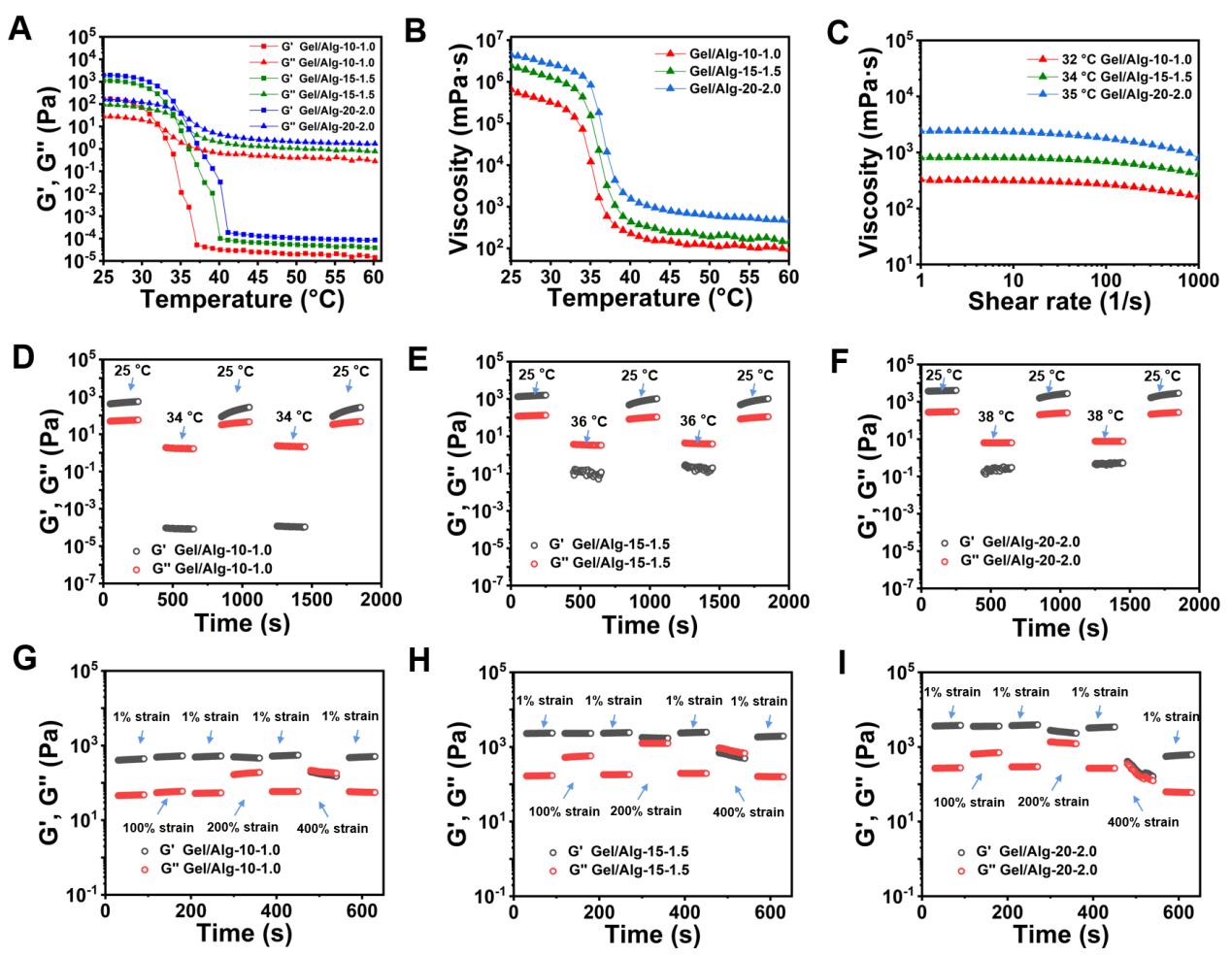


**Fig. S4.** Rheological properties of Gel/Alg inks. (A) Variation of storage modulus (G') and loss modulus (G'') and (B) viscosity of Gel/Alg inks as a function of temperature. (C) Shear-thinning behavior of Gel/Alg inks at their gel-sol transition temperatures. (D-F) Cyclic temperature sweep and (G-I) cyclic strain sweep of Gel/Alg inks (D, G: Gel/Alg-10-1.0; E, H: Gel/Alg-15-1.5; F, I: Gel/Alg-20-2.0).


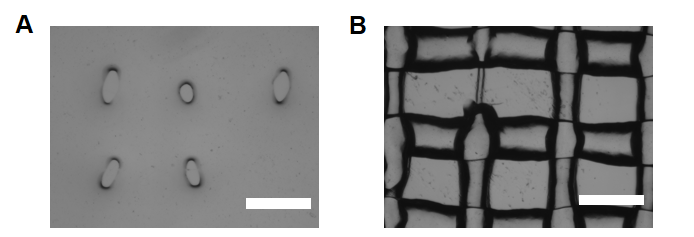


**Fig. S5.** Micrograph of the grid structure printed with Gel/Alg-20-2.0 ink (A) in the under-gel state (printing temperature: 35 ℃) and (B) in the full-gel state (printing temperature: 30 ℃). Scale bar: 750 μm.


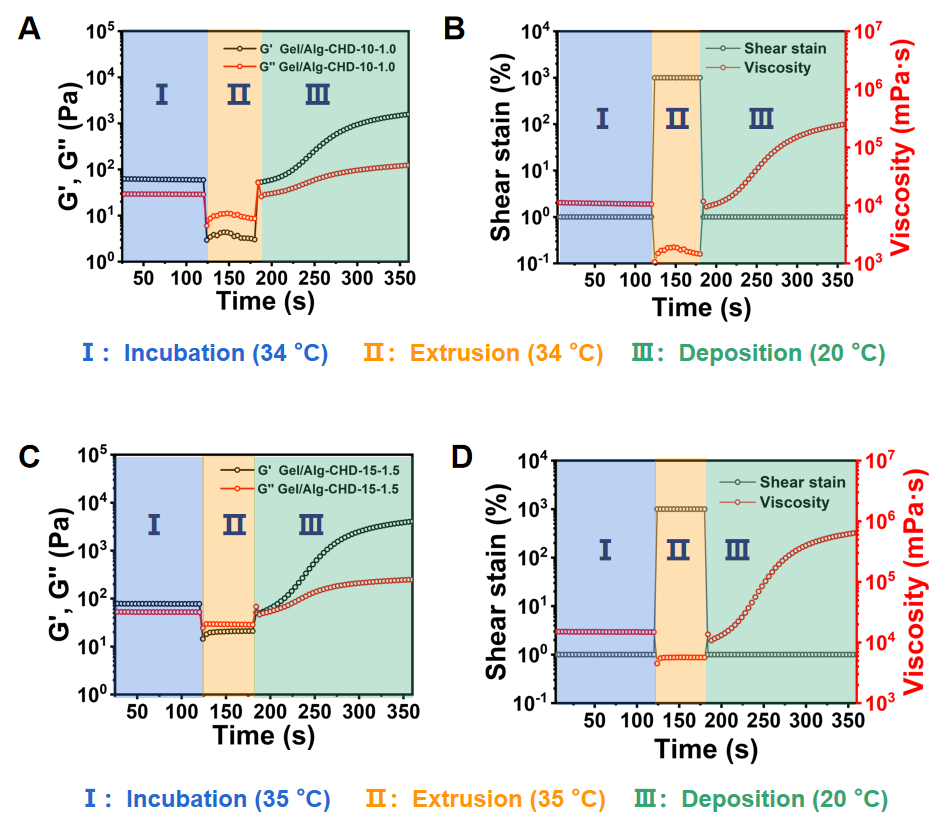


**Fig. S6.** Rheological simulation of the extrusion printing process of (A, B) Gel/Alg-CDH-10-1.0 ink and (C, D) Gel/Alg-CDH-15-1.5 ink. (A , C) The variation of shear modulus (G' and G'') with time; (B, D) The variation of viscosity and shear strain with time.


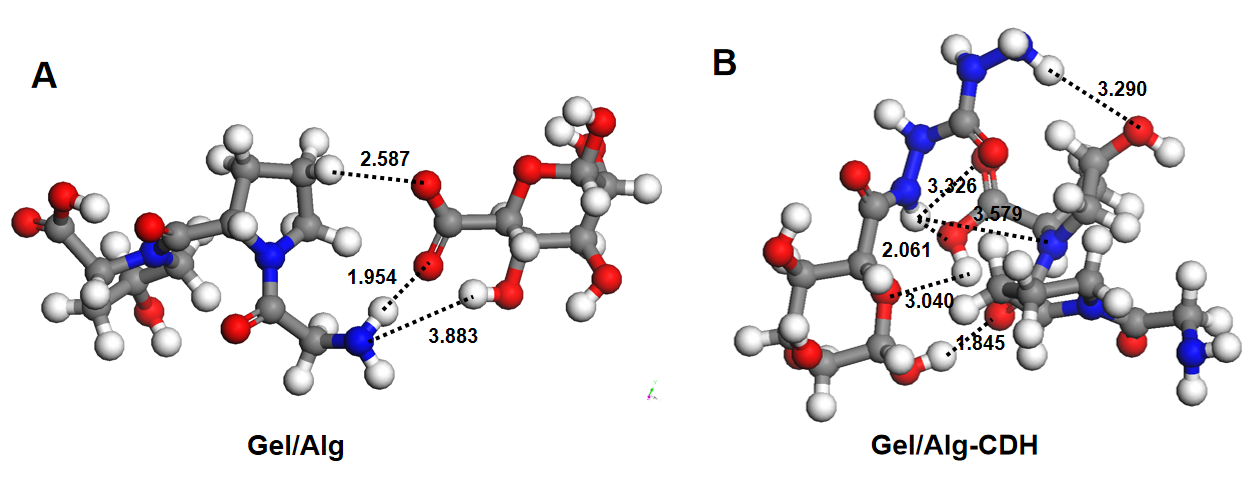


**Fig. S7.** Molecular models were used to calculate the interaction energies among Gel/Alg and Gel/Alg-CDH, in which Gel is simplified to the representative sequence hydroxyproline-proline-glycine (Hyp-Pro-Gly), and Alg is simplified to monosaccharide units. (A) Interaction between Gel and Alg, (B) interaction between Gel and Alg-CDH.


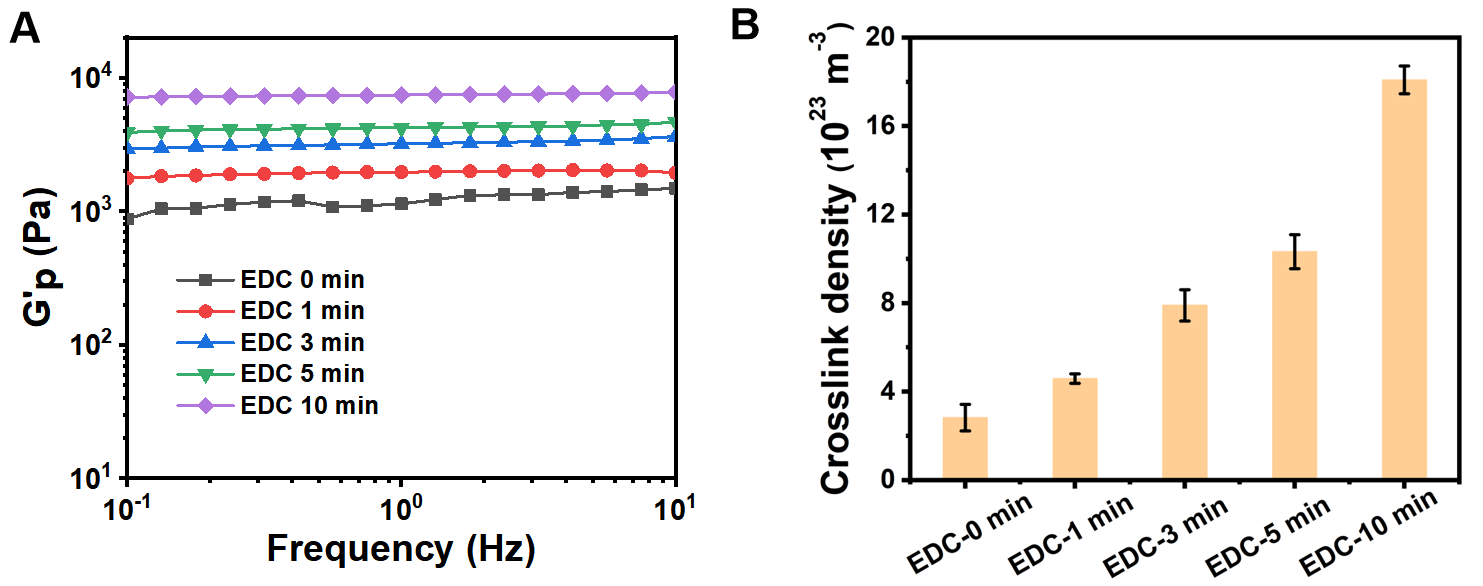


**Fig. S8.** (A) The rubber-elastic plateau G'_p_ of Gel-Alg-CDH-20-2.0-Ca^2+^-EDC hydrogels with different EDC/NHS treatment time. (B) The calculated crosslink density (ν_c_) of Gel-Alg-CDH-20-2.0-Ca^2+^-EDC hydrogels with different EDC/NHS treatment time using the G'_p_.


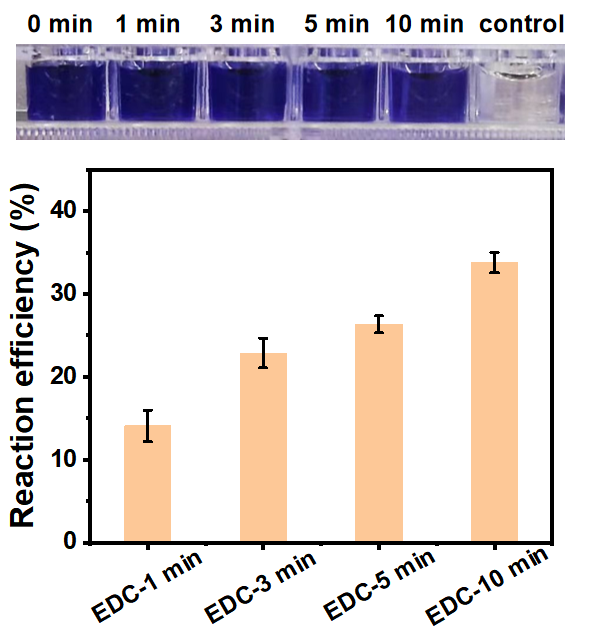


**Fig. S9.** Reaction efficiencies of Gel-Alg-CDH-20-2.0-Ca^2+^-EDC hydrogels with different EDC/NHS treatment time were calculated by determining the amino content using ninhydrine colorimetry.


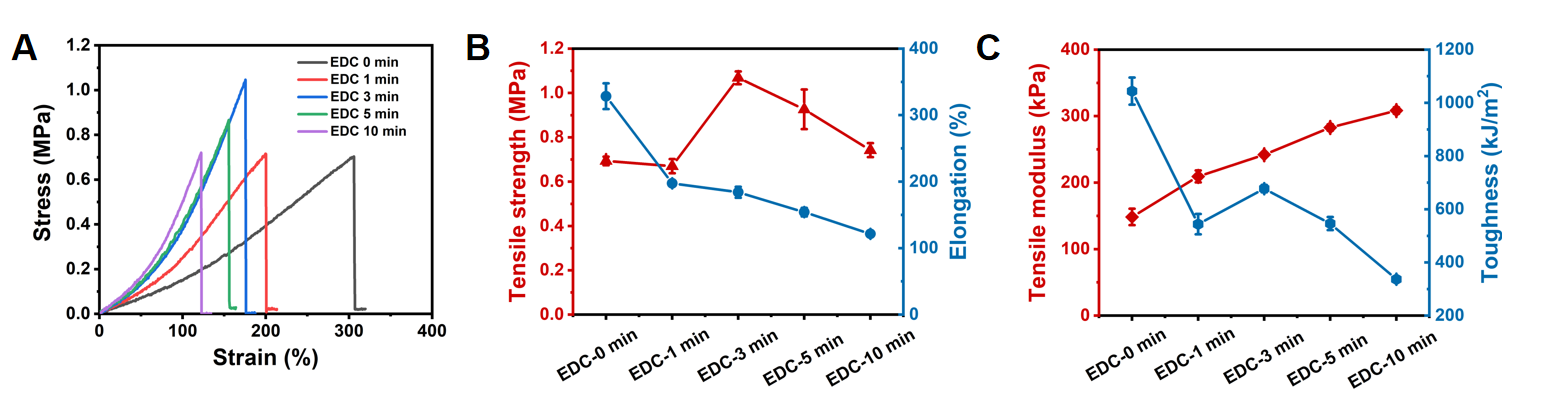


**Fig. S10.** Effect of different EDC/NHS crosslinking treatment time on the mechanical properties of Gel-Alg-CDH-20-2.0-Ca^2+^-EDC hydrogels. (A) Tensile stress-strain curves, (B) tensile strength and elongation at break, (C) tensile modulus (10%-20% strain) and toughness of hydrogels with different treatment times.

**
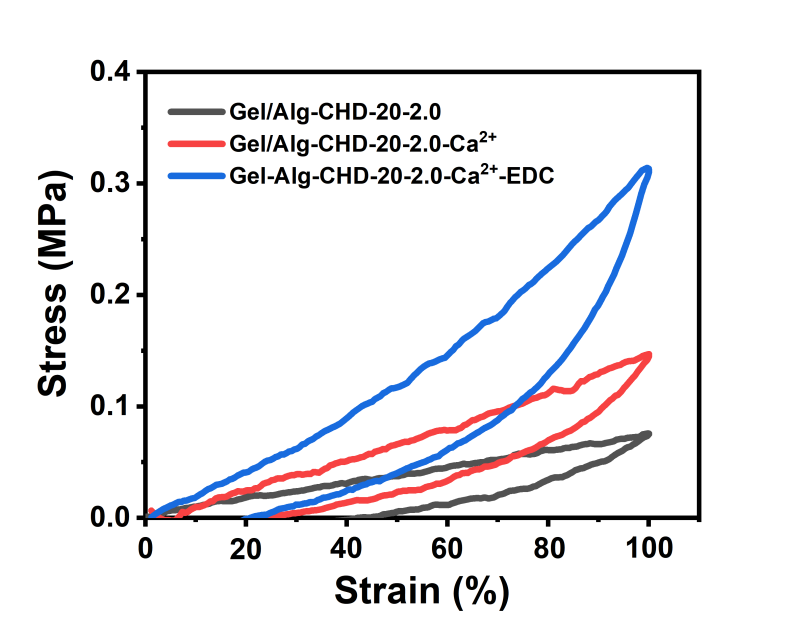
**

**Fig. S11.** Stress-strain curves during loading-unloading process of Gel/Alg-CDH-20-2.0 hydrogel, Gel/Alg-CDH-20-2.0-Ca^2+^ hydrogel and Gel-Alg-CDH-20-2.0-Ca^2+^-EDC hydrogel.


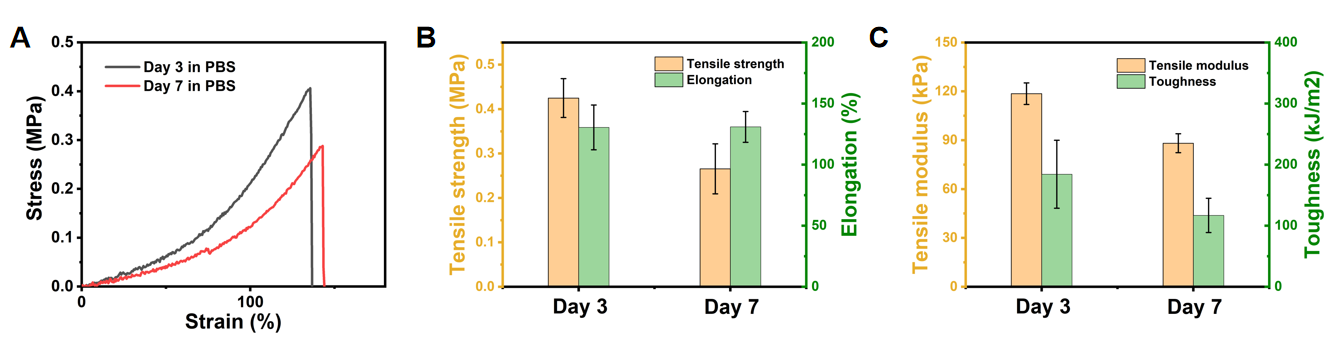


**Fig. S12.** (A) Tensile stress-strain curves, (B) tensile strength and elongation at break, (C) tensile modulus (10%-20% strain) and toughness of Gel-Alg-CDH-20-2.0-Ca^2+^-EDC hydrogel in PBS at 37 ° C for 3 days and 7 days.


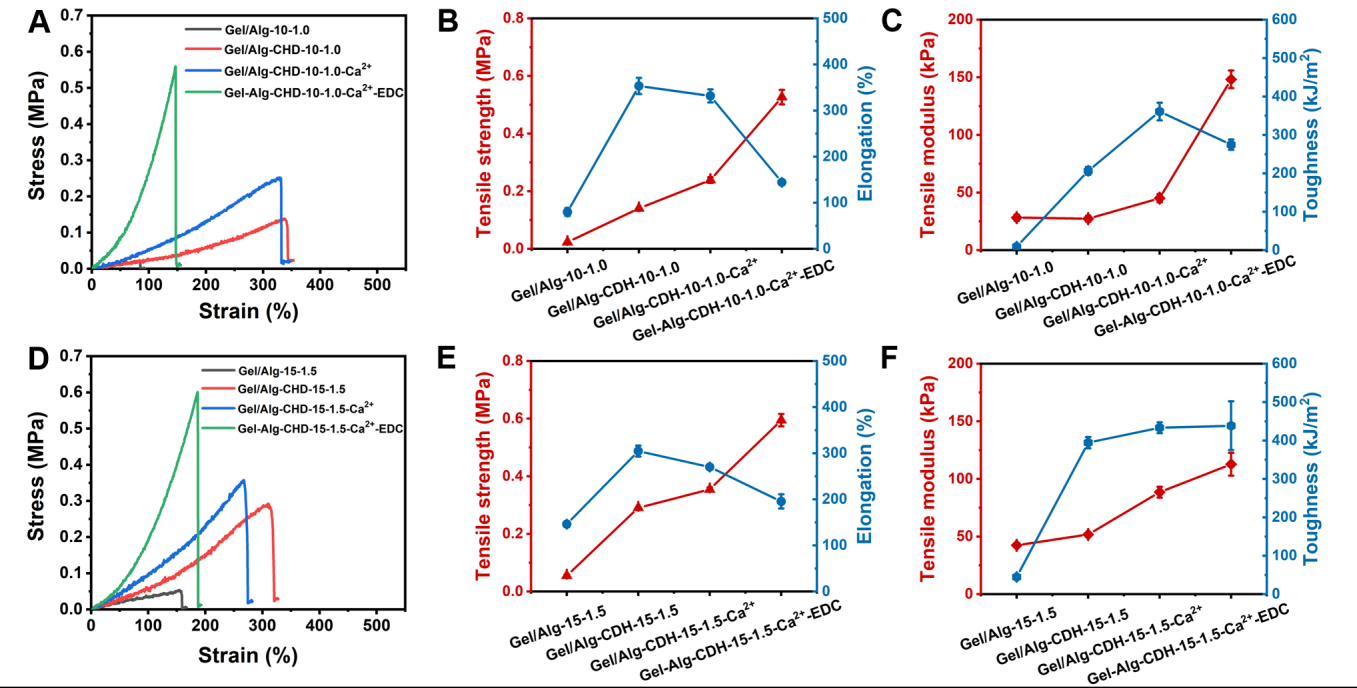


**Fig. S13.** Mechanical properties of (A-C) Gel/Alg-CDH-10-1.0 and (D-F) Gel/Alg-CDH-15-1.5 treated with the sequential strengthening strategy. (A, D) Tensile stress-strain curves, (B, E) tensile strength and elongation at break, (C, F) tensile modulus (10%-20% strain) and toughness of hydrogels treated with the sequential strengthening strategy.


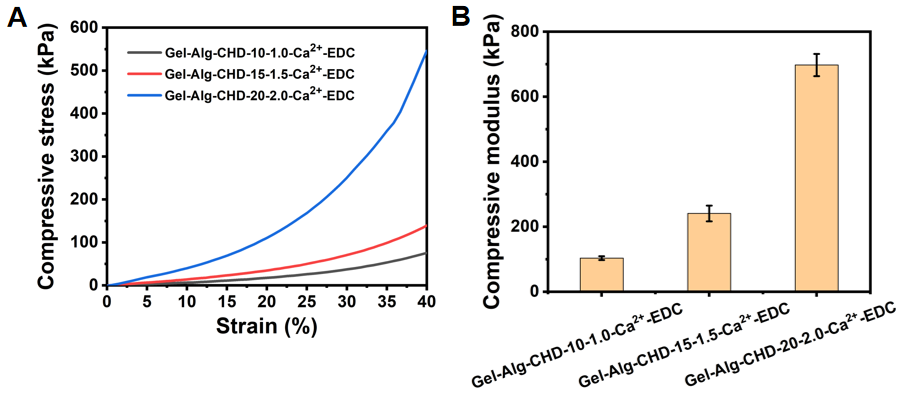


**Fig. S14.** (A) Compressive stress-strain curve and (B) compressive modulus (10% - 20% strain) of Gel-Alg-CDH-10-1.0-Ca^2+^-EDC, Gel-Alg-CDH-15-1.5-Ca^2+^-EDC and Gel-Alg-CDH-20-2.0-Ca^2+^-EDC hydrogel.


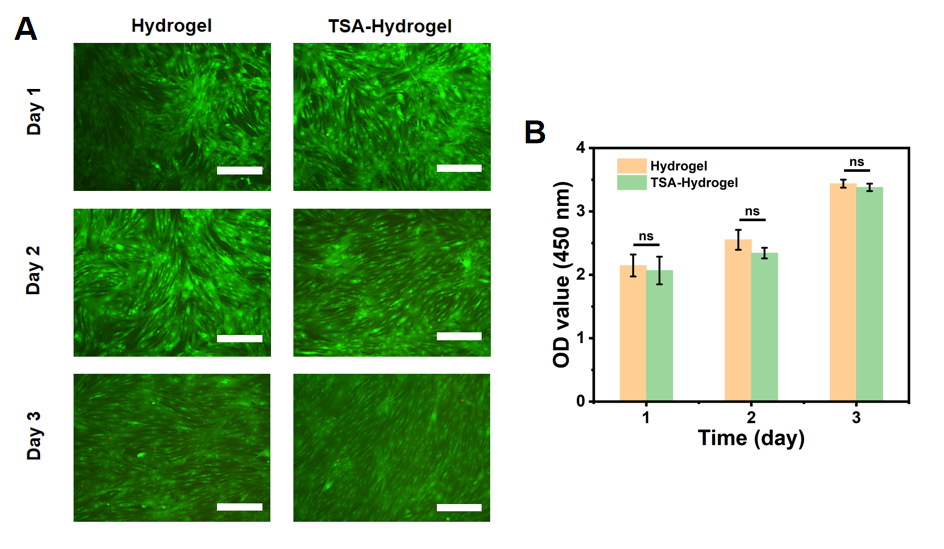


**Fig. S15.** Biocompatibility assessment of TSA-loaded hydrogels. Data are expressed as mean ± standard deviation (n = 4; ns: no significance). (A) Representative Live/Dead Staining images of rASCs treated with hydrogels and TSA-loaded hydrogels for 1, 2, 3 days. Scale bars: 300 μm. (B) CCK-8 assays of rASCs treated with hydrogels and TSA-loaded hydrogel.

**Table S1.** The interaction energies among Gel/Alg and Gel/Alg-CDH calculated by density functional theory.

| Model | Interaction Energy (Kcal/mol) |
| --- | --- |
| Gel/Alg | -24.33 |
| Gel/Alg-CDH | -27.96 |

**Table S2.** The mechanical properties of hydrogels treated with the sequential strengthening strategy .

| Sample | Tensile strength  (MPa) | Elongation  (%) | Tensile modulus  (10%-20% strain)  (kPa) | Toughness  (kJ/m^2^) |
| --- | --- | --- | --- | --- |
| Gel/Alg-10-1.0 | 0.023 ± 0.002 | 79.9 ± 8.7 | 28.2 ± 2.7 | 9.4 ± 1.4 |
| Gel/Alg-CDH-10-1.0 | 0.141 ± 0.002 | 353.4 ± 17.5 | 27.3 ± 2.6 | 206.6 ± 10.2 |
| Gel/Alg-CDH-10-1.0-Ca^2+^ | 0.239 ± 0.010 | 331.8 ± 14.2 | 45.1 ± 3.6 | 361.0 ± 23.0 |
| Gel-Alg-CDH-10-1.0-Ca^2+^-EDC | 0.527 ± 0.025 | 144.3 ± 5.0 | 148.3 ± 7.6 | 274.9 ± 13.5 |
| Gel/Alg-15-1.5 | 0.055 ± 0.004 | 146.1 ± 6.5 | 42.4 ± 0.7 | 44.8 ± 4.3 |
| Gel/Alg-CDH-15-1.5 | 0.291 ± 0.002 | 304.8 ± 11.9 | 51.8 ± 2.0 | 394.5 ± 14.7 |
| Gel/Alg-CDH-15-1.5-Ca^2+^ | 0.354 ± 0.007 | 270.2 ± 3.1 | 88.6 ± 4.7 | 433.5 ± 14.1 |
| Gel-Alg-CDH-15-1.5-Ca^2+^-EDC | 0.595 ± 0.021 | 195.6 ± 15.4 | 112.8 ± 9.9 | 438.6 ± 63.5 |
| Gel/Alg-20-2.0 | 0.130 ± 0.006 | 190.9 ± 18.4 | 97.5 ± 4.3 | 137.6 ± 15.4 |
| Gel/Alg-CDH-20-2.0 | 0.474 ± 0.011 | 406.5 ± 4.5 | 85.6 ± 8.7 | 843.5 ± 8.4 |
| Gel/Alg-CDH-20-2.0-Ca^2+^ | 0.694 ± 0.019 | 328.4 ± 19.4 | 148.5 ± 12.4 | 1044.2 ± 50.9 |
| Gel-Alg-CDH-20-2.0-Ca^2+^-EDC | 1.068 ± 0.029 | 184.1 ± 8.3 | 242.1 ± 3.6 | 677.6 ± 13.7 |
| Gel/Alg-20-2.0-Ca^2+^ | 0.092 ± 0.006 | 184.0 ± 5.0 | 58.5 ± 6.0 | 92.2 ± 6.9 |
| Gel-Alg-20-2.0-Ca^2+^-EDC | 0.315 ± 0.010 | 249.3 ± 12.6 | 82.5 ± 2.9 | 337.1 ± 12.6 |

**Table S3.** Representative thickness of printed bilayer hydrogel scaffolds and the main printing parameters.

| Sample | Air pressure of the bottom layer (MPa) | Air pressure of the top layer  (MPa) | Movement speed  (mm/s) | Path spacing (mm) | Thickness  (μm) |
| --- | --- | --- | --- | --- | --- |
| No.1 | 0.25 | 0.18 | 15 | 0.4 | 250 |
| No.2 | 0.30 | 0.18 | 15 | 0.4 | 300 |
| No.2 | 0.30 | 0.25 | 15 | 0.4 | 400 |

**Movie S1.** The extrusion printing of Gel/Alg-CDH-20-2.0 ink.

**Movie S2.** The printing process of rhEGF/TSA bilayer hydrogel scaffold using Gel/Alg-CDH-20-2.0 ink via a multi-nozzle printing system.

**Movie S3.** The dynamic process of the sutured hydrogel scaffold in withstanding continuous blinking and water flow impact.
